# Daily rhythms in gene expression of the human parasite *Schistosoma mansoni*

**DOI:** 10.1186/s12915-021-01189-9

**Published:** 2021-12-02

**Authors:** Kate A. Rawlinson, Adam J. Reid, Zhigang Lu, Patrick Driguez, Anna Wawer, Avril Coghlan, Geetha Sankaranarayanan, Sarah K. Buddenborg, Carmen Diaz Soria, Catherine McCarthy, Nancy Holroyd, Mandy Sanders, Karl F. Hoffmann, David Wilcockson, Gabriel Rinaldi, Matthew Berriman

**Affiliations:** 1 Wellcome Sanger Institute, Wellcome Genome Campus, Hinxton, UK; 2 King Abdullah University of Science and Technology, Thuwal, Makkah, Saudi Arabia; 3 Institute of Biological, Environmental, and Rural Sciences, Aberystwyth University, Aberystwyth, UK

**Keywords:** Daily rhythms, Transcriptomics, RNA-seq, Adult *Schistosoma mansoni*, Animal circadian clock genes

## Abstract

**Background:**

The consequences of the earth’s daily rotation have led to 24-h biological rhythms in most organisms. Even some parasites are known to have daily rhythms, which, when in synchrony with host rhythms, can optimise their fitness. Understanding these rhythms may enable the development of control strategies that take advantage of rhythmic vulnerabilities. Recent work on protozoan parasites has revealed 24-h rhythms in gene expression, drug sensitivity and the presence of an intrinsic circadian clock; however, similar studies on metazoan parasites are lacking. To address this, we investigated if a metazoan parasite has daily molecular oscillations, whether they reveal how these longer-lived organisms can survive host daily cycles over a lifespan of many years and if animal circadian clock genes are present and rhythmic. We addressed these questions using the human blood fluke *Schistosoma mansoni* that lives in the vasculature for decades and causes the tropical disease schistosomiasis.

**Results:**

Using round-the-clock transcriptomics of male and female adult worms collected from experimentally infected mice, we discovered that ~ 2% of its genes followed a daily pattern of expression. Rhythmic processes included a stress response during the host’s active phase and a ‘peak in metabolic activity’ during the host’s resting phase. Transcriptional profiles in the female reproductive system were mirrored by daily patterns in egg laying (eggs are the main drivers of the host pathology). Genes cycling with the highest amplitudes include predicted drug targets and a vaccine candidate. These 24-h rhythms may be driven by host rhythms and/or generated by a circadian clock; however, orthologs of core clock genes are missing and secondary clock genes show no 24-h rhythmicity.

**Conclusions:**

There are daily rhythms in the transcriptomes of adult *S. mansoni*, but they appear less pronounced than in other organisms. The rhythms reveal temporally compartmentalised internal processes and host interactions relevant to within-host survival and between-host transmission. Our findings suggest that if these daily rhythms are generated by an intrinsic circadian clock then the oscillatory mechanism must be distinct from that in other animals. We have shown which transcripts oscillate at this temporal scale and this will benefit the development and delivery of treatments against schistosomiasis.

**Supplementary Information:**

The online version contains supplementary material available at 10.1186/s12915-021-01189-9.

## Background

Most organisms have biological rhythms that coordinate activities with the consequences of the earth’s daily rotation [1]. These biological rhythms are driven by daily cycles in environmental factors such as temperature, light, predation risk and resource availability, as well as an endogenous molecular circadian clock [2]. Whereas some daily phenotypes are driven by natural environmental cycles and become non-rhythmic in constant conditions, circadian rhythms persist in constant conditions, sustained by an endogenous oscillatory mechanism, the circadian clock. The circadian clock is a molecular network that in animals (Metazoa) is largely conserved across diverse lineages [3, 4]. Interconnected regulatory loops are organised around a core transcriptional-translational feedback loop consisting of the positive factors including *CLOCK* and *ARNTL* (*BMAL1/CYCLE*) and the negative regulators *TIMELESS*, *CRYPTOCHROME* and *PERIOD*, and secondary clock genes that modulate the effects of the core feedback loop; together, these drive oscillations in many clock-controlled genes. Circadian and clock-controlled genes are a subset of the genes that show daily patterns of expression (diel genes), and all together they lead to 24-h patterns in physiology and behaviour.

Much like their free-living counterparts, some parasites living within the bodies of other organisms are known to have biological rhythms. In the case of a malaria-causing parasite, *Plasmodium chabaudi*, these rhythms maximise its fitness in terms of within-host survival and between-host transmission [5]. Understanding the rhythms of parasites will provide insight into how they temporally compartmentalise their internal processes and host interactions to survive the daily cycles of the host immune system and physiology. Furthermore, understanding both parasite and host rhythms may enable the development of vaccines and drugs that take advantage of rhythmic vulnerabilities in parasites or harness host rhythms to improve efficacy and reduce drug toxicity [6]. Recent work on blood-dwelling protozoan parasites has revealed daily rhythms in gene expression, physiology, drug sensitivity and the presence of an intrinsic clock [7–9]. However, similar studies on metazoan parasites are lacking. Exploring daily molecular oscillations in metazoan parasites will give us insight into how these longer-lived organisms can survive host daily cycles over a lifespan of many years, and will lead to an understanding of how circadian clockwork has evolved in parasitic animals.

One long-lived metazoan parasite is *Schistosoma mansoni*, a blood-dwelling flatworm (Platyhelminthes), that can live in the human vasculature for over 30 years [10]. It causes schistosomiasis, a major neglected tropical disease, that has a profound human impact, with an estimated 140,000 cases and 11,500 deaths in 2019 [11]. Nothing is known about whether the adult worms (which give rise to the pathology-causing eggs) exhibit any daily or circadian rhythms in any aspect of their biology because they live deep inside the portal veins. Earlier in the development, *S. mansoni* cercariae larvae are shed from the snail host at a population-specific time of day [12], but the molecular underpinnings of this rhythm are not known. *S. mansoni* naturally infects both humans and rodents in the wild (rats—*Rattus rattus* and *Arvicanthis niloticus*, and mice—*Mastomys huberti* [12, 13]), and the mouse (*Mus musculus*) is commonly used as the definitive host in the laboratory maintenance of its life cycle. The mouse, *M. musculus*, is also the model species routinely used to study circadian rhythms in mammals [14], and because of this, we know there are many daily rhythmic fluctuations in the vasculature (e.g. temperature, pressure, oxygen, glucose, red and white blood cells [15–19]) that may act as zeitgebers (German for “time giver” or synchroniser) to influence the worm’s biology and potentially its rhythms.

Here, we first asked whether sexually mature male and female *S. mansoni*, collected from their natural environment (the mesenteric vasculature of mice, under ‘normal’ host conditions—a 12:12-h light:dark cycle), have daily rhythms in their transcriptomes. We investigated the possible function of the diel genes and determined whether canonical metazoan circadian clock genes are present and rhythmic and may therefore play a role in these daily rhythms. Our discovery that gene expression oscillated throughout the 24-h period, and our investigation into the putative function of these diel genes, has given us an understanding of the fine-scale temporal partitioning of biological processes in male and female worms, including indications of parasite/host interactive daily rhythms. Finally, we identified potential drug targets and vaccine candidates amongst the diel genes and found existing drugs that are predicted to interact with diel gene products. The implication that timing of target expression could affect the efficacy of drugs or vaccines is an important consideration for the development and delivery of new interventions against schistosomiasis.

## Results

**Fig. 1:**
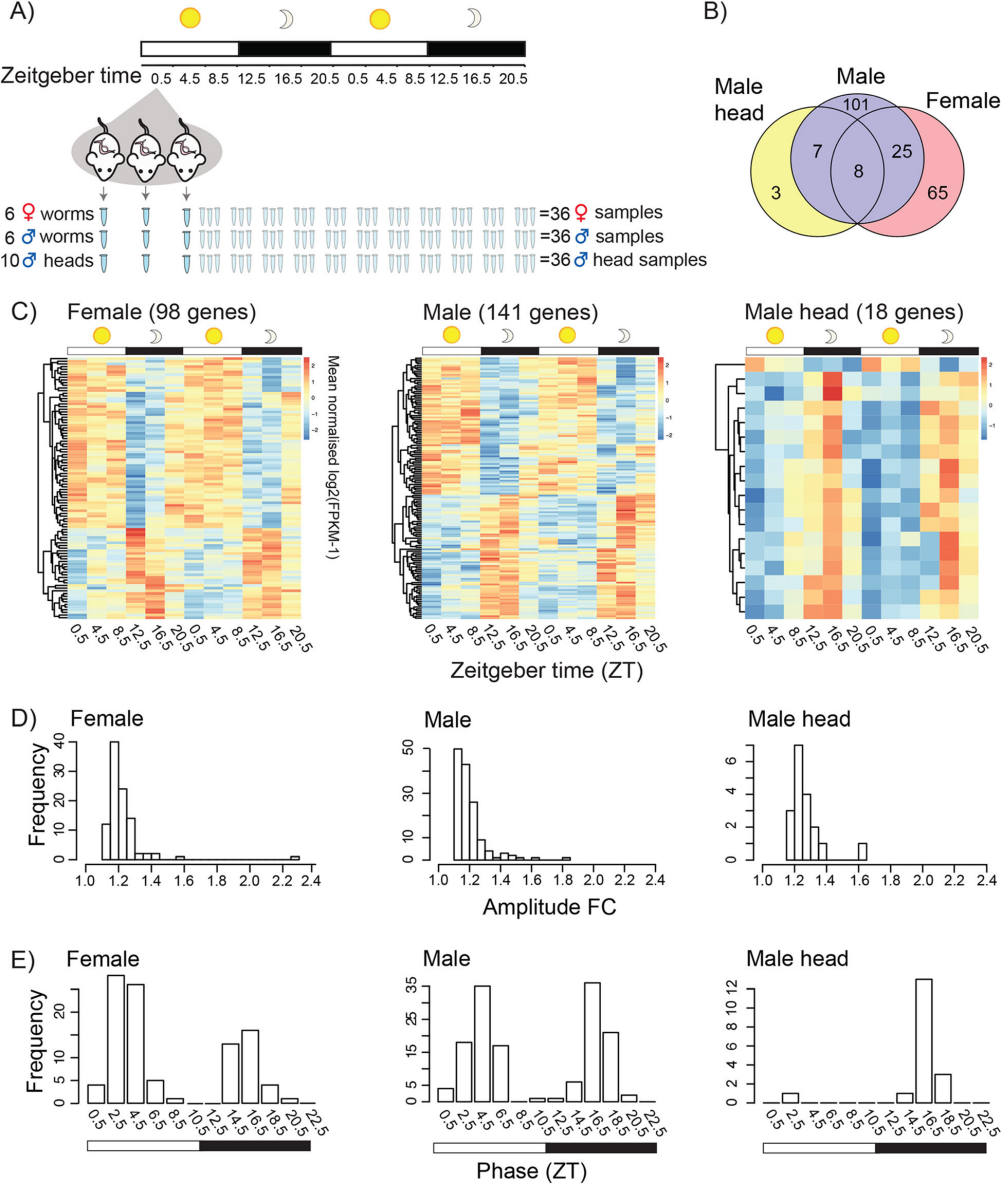
Twenty-four-hour periodicity in the transcriptomes of adult *Schistosoma mansoni*. **A** Schematic showing the collection of pooled worm samples from three mice every 4 h over 44 h, used to generate three RNA-seq time-series datasets: one for whole female worms, once for whole male worms and one for male heads. **B** In total, 209 diel genes were identified in *S. mansoni* (JTK_Cycle BH.Q < 0.01), with some showing 24-h periodicity in more than one dataset. **C** Expression heatmaps of diel genes. Each row represents a gene whose transcripts oscillate with ∼24 h periodicity, ordered vertically by phase. **D** Histograms of rhythmic daily fold changes (FC) in transcript abundance. **E** Histograms showing the distribution in peaks of expression in diel genes in both sexes and in male heads

We collected mature female and male whole worms, as well as the heads of mature males (to enrich for the cephalic ganglia or brain; the site of the master circadian clock in some animals), at 4-h intervals over a 44-h period from mice entrained in alternating 12-h light and dark cycles (LD12:12). Although light is probably not a relevant zeitgeber to schistosome adults (as light penetration beyond 5 mm is minimal in mammalian bodies [20]), light is an important cue for the mouse, and as nocturnal creatures, they are active mainly during the dark phase [21]. Zeitgeber time (ZT) 0 indicates the beginning of the light phase and resting phase for the mouse (which we call day-time here), and ZT 12 is the beginning of the dark phase and active phase for the mouse (called night-time in this study). Male and female worms from each mouse were separated from each other and pooled by sex. Heads of mature male worms were isolated, and these were pooled into a sample for each mouse. RNA was extracted from each pool and sequenced. This gave us three time-series datasets: one for whole female worms, one for whole males and one for male heads. In each dataset, three biological replicates per time point were collected with each replicate being a sample of pooled worms (or worm heads) from one mouse (Fig. 1A).

### There are daily rhythms in the transcriptomes of adult *Schistosoma mansoni*

**Fig. 2:**
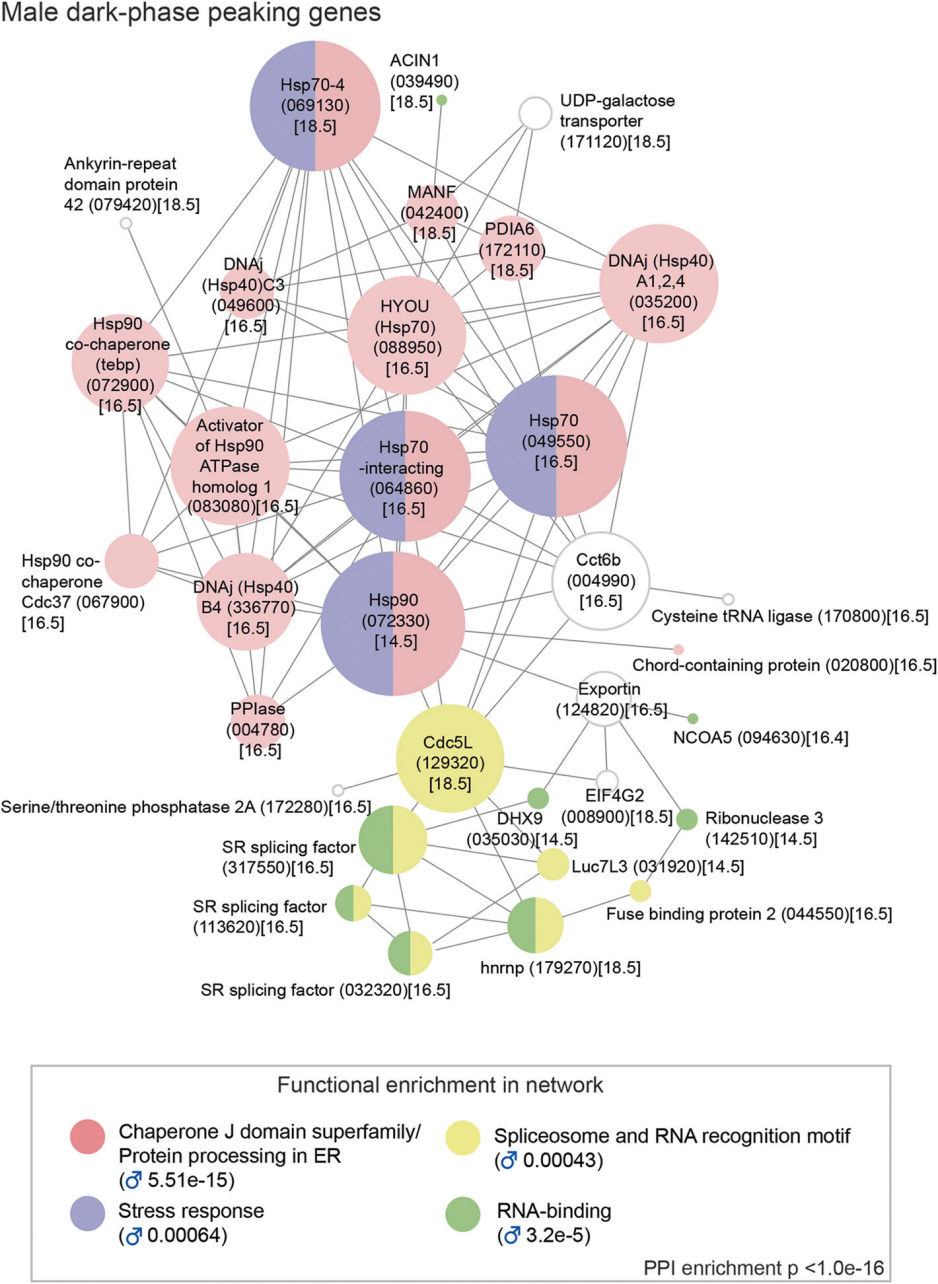
Dark-phase peaking transcripts in males form a network of heat shock and RNA-binding/spliceosome genes*.* Predicted molecular interaction network for genes encoding transcripts found to peak during the night-time/dark phase/host active phase were computed using the STRING online database. All peaked between 14.5 and 18.5 zeitgeber time (ZT) (i.e. between 2.5 and 6.5 h after the start of the dark phase). Node size reflects the number of connections a molecule has within the network. Lines (edges) connecting nodes are based on evidence of the function of homologues. Functional enrichment (*FDR*) as provided by STRING. (PPI = predicted protein interaction; gene identifiers shown in parenthesis but with “Smp_” prefix removed for clarity; acrophase/time of peak expression (ZT) in square brackets)

**Fig. 3:**
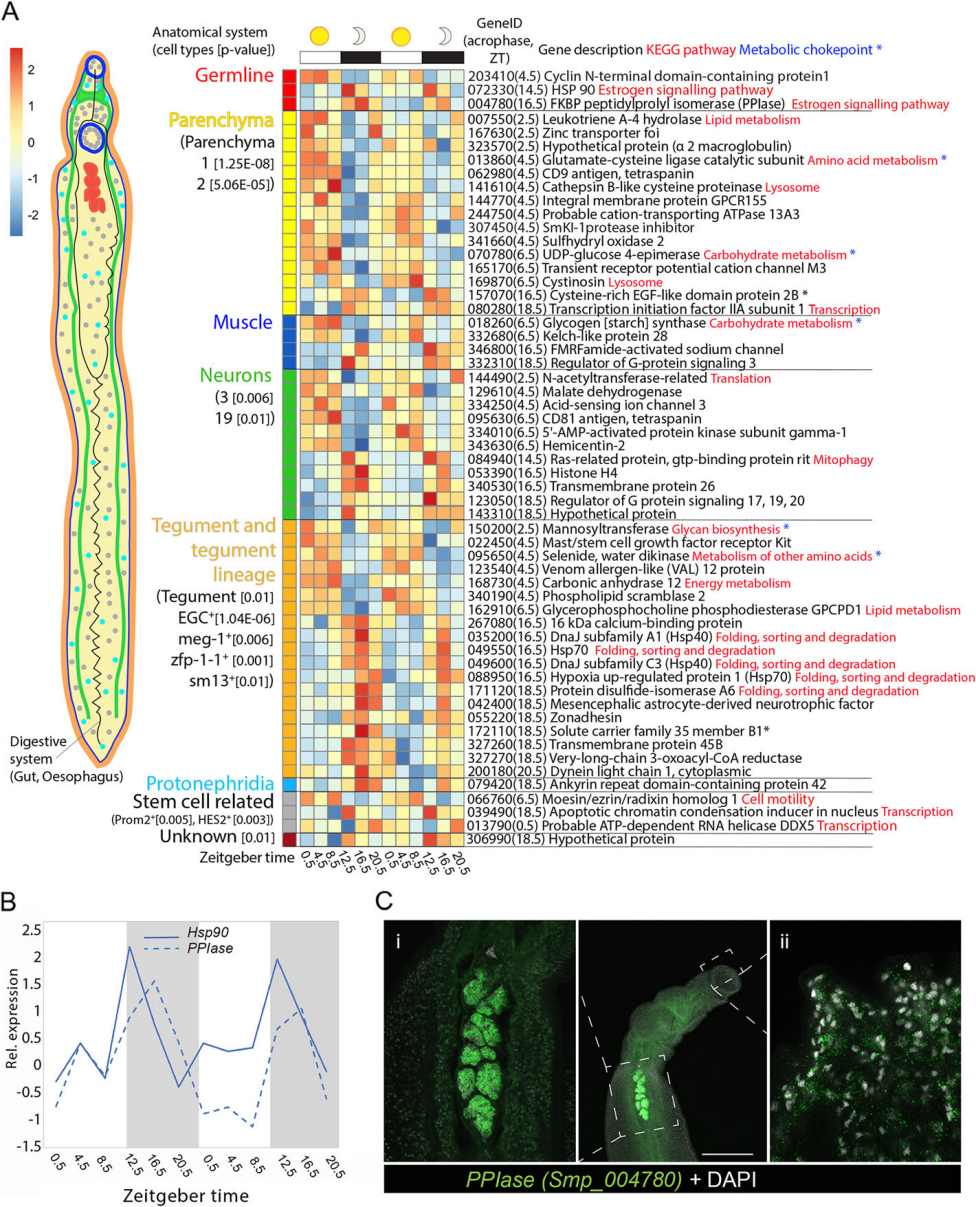
Anatomical and cell-type context of diel genes in male worms. **A** Heatmap of the 57 male diel genes that are single cell markers [25]. Each row represents a diel gene, ordered vertically by phase within anatomical systems containing constituent cell types. Diel genes that are markers for multiple cell type categories are placed within cell type (and their category) with the greatest difference between the Seurat pct1-pct2 scores. “Smp_” prefixes have been removed from gene identifiers for clarity; time of peak expression (acrophase) in parentheses (ZT = Zeitgeber time). *p* value of cell types significantly enriched in diel genes given in brackets [*p* < 0.05]. **B** Temporal relative expression profiles of *Hsp90* (Smp_072330) and *FKBP-type peptidylprolyl isomerase* (*PPIase*) (Smp_004780) showing peaks of expression at night. **C** WISH expression of *PPIase* showing labelled transcripts in male worm (i) throughout the testes and (ii) in the head (scale bar = 100 μm) (100% of individuals examined, *n* = 10)

The mean number of expressed genes in all samples across the time series in female, male and male heads datasets was 8312, 8365 and 8475, respectively (81.7, 82.2 and 83.3% of genes in *S. mansoni* version 7; Additional file 1: Table S1-3). From each dataset, we identified genes that were differentially expressed (false discovery rate [*FDR*] < 0.05) over a 24-h period: 206 in females, 194 in males and 48 in male heads (Additional file 1: Table S4-6 [22]). For these, ~ 24-h periodicity was identified using JTK_Cycle (Jonckheere-Terpstra-Kendall) (Benjamini-Hochberg *q* values [BH.Q] < 0.05) (Additional file 1: Table S4-6 [23, 24]) but we present only those with JTK BH.Q < 0.01, i.e. those that have the most significant fit to the symmetric sine-waveform of JTK. Using this strict threshold, we found 98 diel genes in females, 141 in males and 18 in male heads, corresponding to 1.2%, 1.7% and 0.2% of the transcriptomes, respectively (Fig. 1B, C; Additional file 1: Table S4-7). The combined number of diel genes from the three datasets was 209, 2.1% of the genome (Fig. 1B; Additional file 1: Table S7). A significant number of diel genes were shared between males and females (33; Fisher’s exact test odds ratio = 47, *p* < 10^−16^) and between males and male heads (15; Fisher’s exact test odds ratio = 395, *p* < 10^−16^; Fig. 1B, Additional file 1: Table S7). The median peak-to-trough fold change (amplitude) of gene expression for diel genes was 1.19, 1.18 and 1.24 for females, males and male heads, respectively. However, many genes had much higher daily fold changes in expression (Fig. 1D; Table 1); the highest in each of the datasets were *SmKI-1* (Smp_307450, fold change 1.83) in males that encodes a BPTI/Kunitz protease inhibitor domain protein, as well as *hsp70* (Smp_049550, 1.63 fold) in male heads and *hsp90* (Smp_072330, 2.30 fold) in females that each encode heat shock proteins (HSPs) (Table 1; see Additional file 1: Table S8 for orthologs in other taxa**)**. In males, *Hsp90* has a 4.8-fold change; it falls just outside the strict JTK BH.Q < 0.01 threshold [JTK BH.Q = 0.0103] but we include it in downstream analyses (below, and Figs. 2 and 3). While we identified rhythmic genes with peaks of expression at most times of day, there was a clear bimodal pattern in both females and males (Fig. 1E). In females, expression peaked at 2.5–4.5 h (ZT) after lights on and at 14.5–16.5 h (ZT) (2.5–4.5 h after lights off), with 66% of the cycling genes peaking during the day (the hosts resting phase), while in males the peaks occurred at 4.5 and 16.5 (ZT) (with a 53:47% split between light:dark phases). In the heads of males, the expression of diel genes peaked at ZT 16.5 and was not bimodal, with all but one reaching peak expression during the night (the hosts active phase).

**Table 1:** The ten diel genes with the highest amplitudes (daily fold change) in females, males and male heads

Dataset	Gene ID	Gene description	Daily fold change	Phase (ZT)	JTK BH.Q
Female	Smp_072330	Heat shock protein 90	2.30	**14.5**	0.00032
	Smp_326610	Trematode eggshell synthesis domain-containing protein	1.56	6.5	0.00191
	Smp_004780	FKBP-type peptidylprolyl isomerase (PPIase)	1.41	**16.5**	0.00019
	Smp_044850	Ribokinase	1.41	**16.5**	0.00770
	Smp_342000	FMRFamide-activated amiloride-sensitive sodium channel	1.38	8.5	0.00191
	Smp_069130	Heat shock protein 70 (Hsp70)-4	1.35	**14.5**	0.00029
	Smp_319380	n/a	1.33	2.5	0.00362
	Smp_244190	ZP domain-containing protein	1.31	**14.5**	0.00588
	Smp_340010	Putative eggshell protein	1.30	4.5	0.00235
	Smp_064860	Putative heat shock protein 70 (Hsp70)-interacting protein	1.30	**16.5**	7.92E−05
Male	Smp_307450	SmKI-1, a BPTI/Kunitz protease inhibitor domain protein	1.83	4.5	0.00311
	Smp_049550	Putative heat shock protein 70 (Hsp70)	1.62	**16.5**	1.21E−06
	Smp_324960	Hypothetical protein	1.57	**18.5**	0.00112
	Smp_327270	Very-long-chain 3-oxoacyl-CoA reductase	1.52	**18.5**	0.00763
	Smp_327260	Transmembrane protein 45B	1.49	**18.5**	0.00468
	Smp_013950	Solute carrier family 43 member 3	1.48	0.5	0.00112
	Smp_328570	n/a	1.48	4.5	0.00763
	Smp_327240	Hypothetical protein	1.45	**18.5**	0.00651
	Smp_004780	FKBP-type Peptidylprolyl isomerase (PPIase)	1.36	**16.5**	7.31E−05
	Smp_013790	Probable ATP-dependent RNA helicase, DDX5	1.35	0.5	0.00047
Male head	Smp_049550	Putative heat shock protein 70 (Hsp70)	1.63	**16.5**	1.43E−05
	Smp_004780	FKBP-type peptidylprolyl isomerase (PPIase)	1.35	**16.5**	0.00055
	Smp_145560	Monocarboxylate transporter 9	1.33	2.5	0.00236
	Smp_124820	Putative chromosome region maintenance protein 1/exportin	1.30	**18.5**	0.00236
	Smp_069130	Heat shock protein 70 (Hsp70)-4	1.30	**16.5**	0.00236
	Smp_333170	85/88 kDa calcium-independent phospholipase A2	1.28	**18.5**	0.00188
	Smp_113620	Serine/arginine-rich splicing factor 2	1.27	**16.5**	0.00297
	Smp_049600	Putative DNAj (Hsp40) homologue, subfamily C, member 3	1.25	**16.5**	7.31E−05
	Smp_214080	VEZF1/Protein suppressor of hairy wing/Zld/ C2H2-type	1.25	**16.5**	7.49E−06
	Smp_246230	Nicotinate-nucleotide pyrophosphorylase	1.24	**16.5**	0.00035

The ten diel genes with the greatest fold change in expression over a 24-h period were identified by ranking the diel genes by fold change in each dataset. Their daily fold change, peak phase of expression (zeitgeber time, ZT, bold = night) and significance of 24-h rhythmicity determined using JTK_Cycle are presented

### Diel genes have distinct night-time and day-time processes

Our annotation-enrichment analyses showed that the diel genes are involved in distinct rhythmic processes during the night and day-time, and we present below night-time and day-time peaking genes separately, highlighting similarities and differences between the sexes during each period.

To gain further insight into the putative function of diel genes, we also identified those that are markers of the 68 cell types identified in adult *S. mansoni* [25, 26].

#### Night-time peaking genes (host active phase)

Of the 66 genes with a night-time peak in males, 33 formed a single large network based on their predicted STRING interactions (Fig. 2). Within this network, many of the molecules with the largest number of interactions were putative molecular chaperones (orthologs to heat shock proteins and their co-chaperones) involved in protein processing in the ER (endoplasmic reticulum) and the stress response. In females, a smaller network of 13 genes was predicted, but heat shock/stress response genes were still the main constituents (Additional file 2: Fig. S1). These observations were supported by enrichment of GO (Gene Ontology) terms related to protein folding and chaperones: protein folding/unfolded protein binding (males *FDR* 0.0013, females *FDR* 10^−9^; Additional file 1: Table S9). Diel genes involved in these processes and networks all had acrophases between 14.5 and 18.5 ZT (i.e. mid-dark phase; Additional file 2: Fig. S2) and could be mapped to three KEGG (Kyoto Encyclopedia of Genes and Genomes) pathways: ‘protein processing in the ER’ (Additional file 2: Fig. S3), ‘PI3K-AKT signalling’ (Additional file 2: Fig. S4) and ‘Estrogen signalling’ (Additional file 2: Fig. S5). Orthologs of six of the diel HSPs in *S. mansoni* show 24-h rhythms in other animals as well (Additional file 1: Table S7, 8 & 16).

**Fig. 4:**
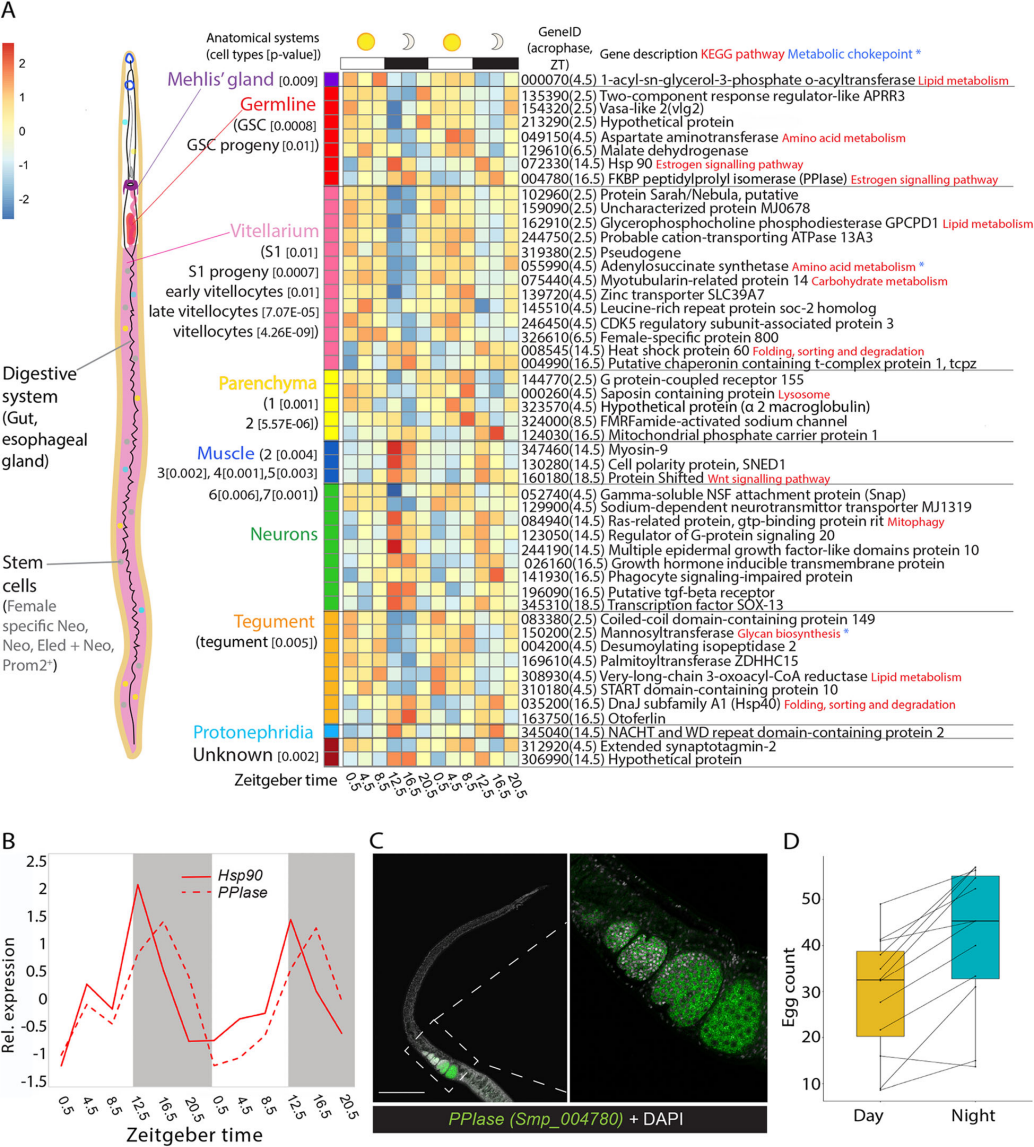
Anatomical and cell-type context of diel genes in female worms. **A** Heatmap of the 48 female diel genes that are single cell markers [25]. Each row represents a diel gene, ordered vertically by phase within anatomical systems containing constituent cell types. Diel genes that are markers for multiple cell type categories are placed within cell type (and their category) with the greatest difference between their Seurat pct1-pct2 scores. “Smp_” prefixes have been removed from gene identifiers for clarity; time of peak expression (acrophase) in parentheses (ZT = Zeitgeber time). *p* value of cell types significantly enriched in diel genes given in brackets [*p* < 0.05]. **B** Temporal relative expression profiles of *Hsp90* (Smp_072330) and *FKBP-type peptidylprolyl isomerase* (*PPIase*) (Smp_004780) showing peaks of expression at night. **C** WISH expression of *PPIase* showing labelled transcripts in female worms in all oocytes in the ovary (scale bar = 100 μm) (100% of individuals examined, *n* = 10). **D** Female (paired) worms in vitro lay more eggs at night (dark phase) (median and interquartile ranges) than during the day (*n* = 12 female worms; median (night egg count − day egg count) = 12.0; paired Wilcoxon test: *p* = 0.003216)

Ten HSPs and co-chaperones cycled in both sexes, with eight oscillating in phase synchrony between the sexes (Additional file 2: Fig. S2). A striking example of this was seen in *hsp90* (Smp_072330) and *FKBP-type peptidylprolyl isomerase* (*PPIase*) (Smp_004780); *hsp90* peaks at the start of the dark phase, 4 h before *PPIase* (Figs. 3 and 4). Although their functions are not known in *S. mansoni*, both are single cell markers for germ stem cell progeny and we show, using whole-mount in situ hybridisation (WISH), that *PPIase* is expressed in sperm throughout the testes and in all oocytes in the ovary (Fig. 3C and 4C); *PPIase* also cycles in male head samples and is expressed in many cells in the head (Fig. 3C). There were also sex differences in the diel genes involved in the nightly chaperone response. For example, a *DNAj* (*hsp40*), which has homologues located on the different sex chromosomes (known as gametologues) [27], has diel expression in both gametologues. Schistosomes have a ZW sex chromosome system, where females are ZW and males are ZZ. The Z gametologue (Smp_336770) cycles in females, males and male heads, and the W gametologue (Smp_020920) also cycles, and is present only in females (Additional file 2: Fig. S2). Further sex differences included diel expression, in males only, of nine genes whose orthologs are involved in stress response and recovery (Additional files 2: Fig. S2). Four of these genes are markers for tegument-related cell types (Fig. 3) and as the male tegument forms a large surface area that is in direct contact with mouse blood and endothelium, these cells are likely to be exposed to environmental heat shock triggers. Night-time peaking genes were also associated with GO terms related to RNA binding and mRNA splicing, but in males only (FDR = 0.0002 for RNA binding, FDR = 0.0292 for mRNA splicing; Additional file 1: Table S9). Based on STRING, many of these genes form part of the night-time network (Fig. 2), including putative homologues of *human Ser/Arg-rich splicing factors* (Smp_317550, Smp_032320, Smp_113620); *heterogeneous nuclear ribonucleoprotein* (Hnrp; Smp_179270): the *cell division cycle control protein Cdc5L* (Smp_129320), a spliceosome component; and the splicing regulator [28] *far upstream element-binding protein* (Smp_044550). The night-time network predicts an interaction between heat shock proteins and RNA-binding and mRNA splicing genes, connected via *Cdc5L* (Fig. 2).

Diel genes involved in the regulation of G-protein-coupled receptor functioning (*regulator of G-protein signalling 3 [RGS3]* Smp_332310; and *RGS20* Smp_123050) peaked at night and are markers for muscle and nerve cells, respectively (Figs. 3 and 4). *Histone 4* (Smp_053390) and *gtp-binding Ras-related protein* (Smp_084940), a member of the histone co-chaperone pathway, also peaked at night in males and are markers for nerve cell types (Fig. 3). Neuronal activity promotes histone turnover [29, 30], and taken together, these results may indicate higher sensory responsiveness and activity at night.

#### Day-time peaking genes (host resting phase)

**Fig. 5:**
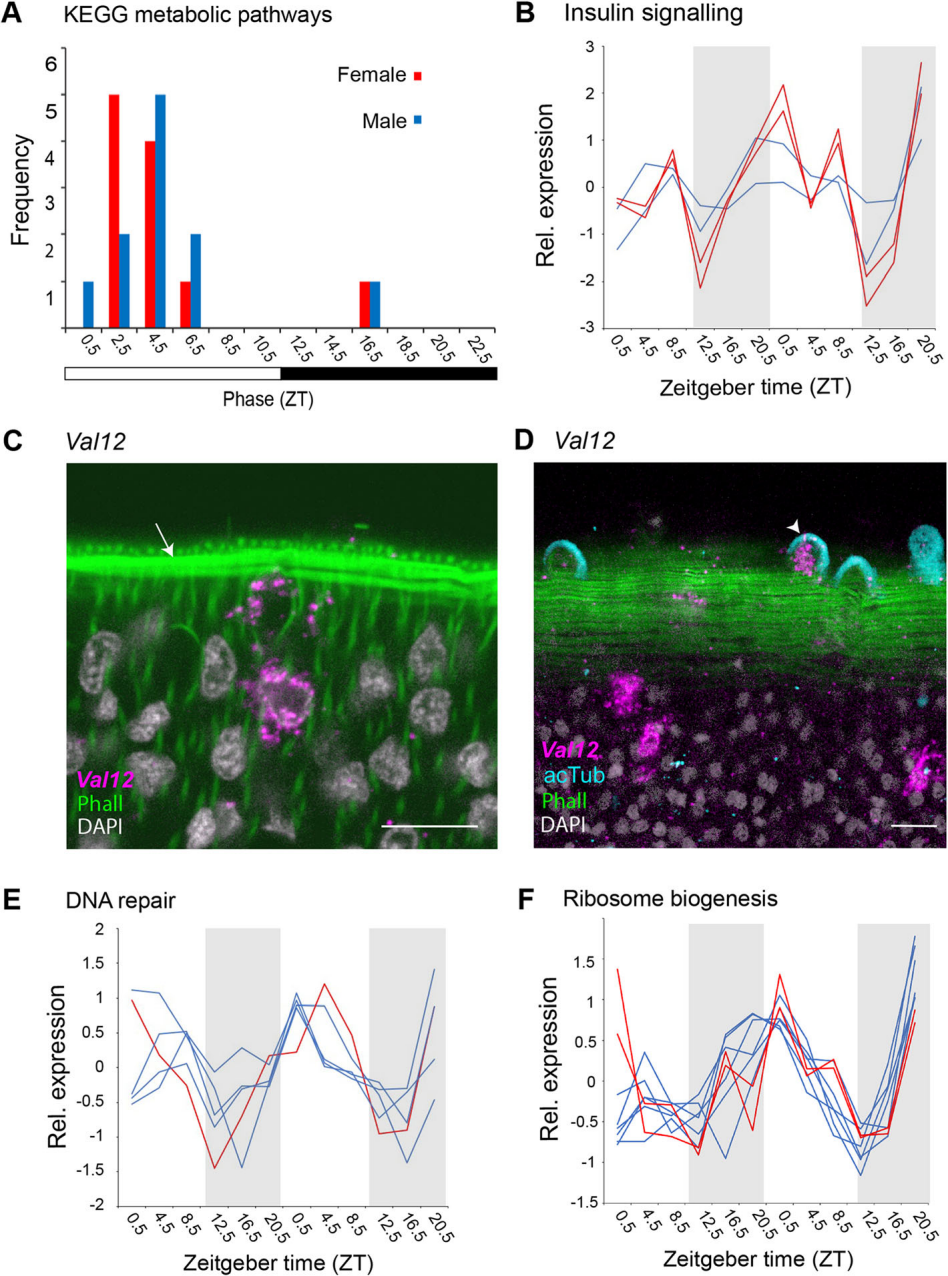
Light-phase peaking diel genes are involved in metabolism, host interaction, DNA repair and ribosome biogenesis. **A** Peak phase of expression of diel genes in KEGG pathways for metabolism. **B** Temporal expression profiles of genes involved in the insulin signalling pathway. **C**, **D** *Venom-allergen-like 12* (*Val12*, Smp_123540) is a diel gene in males; it peaks at noon and is predicted to interact with the host. In situ expression supports this prediction as transcripts reach the body wall musculature (arrow) and into the tubercles of the dorsal tegument (arrow head) (scale = 10 μm, optical sections, phall = phalloidin, acTub = acetylated tubulin, 100% of individuals examined, n = 20). **E** Temporal expression profiles of genes involved in DNA repair and **F** ribosome biogenesis (**B**, **E** and **F** blue and red lines = male and female diel genes respectively)

Predicted interactions between the day-time peaking genes were more limited than at night; both sexes had interactions of genes associated with metabolism (Additional file 2: Fig. S6). There were 32 diel genes that could be mapped to KEGG metabolic pathways; most of which are involved in lipid, carbohydrate and amino acid metabolism (Additional file 1: Table S11). Of these, 28 have acrophases between 0.5 and 6.5 ZT, suggesting a peak in metabolic activity at this time: an extended metabolic ‘rush hour’ (Fig. 5A). We identified 21 diel genes that have previously been classified as metabolic chokepoints (capable of uniquely generating specific products or utilising specific substrates [31]) for potentially targeting with new drugs; 15 of these peaked during the day (Additional file 1: Table S7). Several metabolic diel genes were also markers of specific cell types: those of the reproductive system in female worms and those of tegumental, parenchymal and muscle cells in males (Figs. 3 and 4). There are four diel genes involved in the insulin signalling pathway and they all peaked between 2.5 and 6.5 ZT: *glycogen synthase* (Smp_018260) and *3-phosphoinositide-dependent protein kinase 1* (Smp_094250) in males and *hexokinase* (Smp_043030) and *protein phosphatase 1 regulatory subunit 3B* (Smp_167660) in females (the latter three had JTK BH.Q values of 0.01–0.05) (Additional file 1: Table S11, Fig. 5B).

**Fig. 6:**
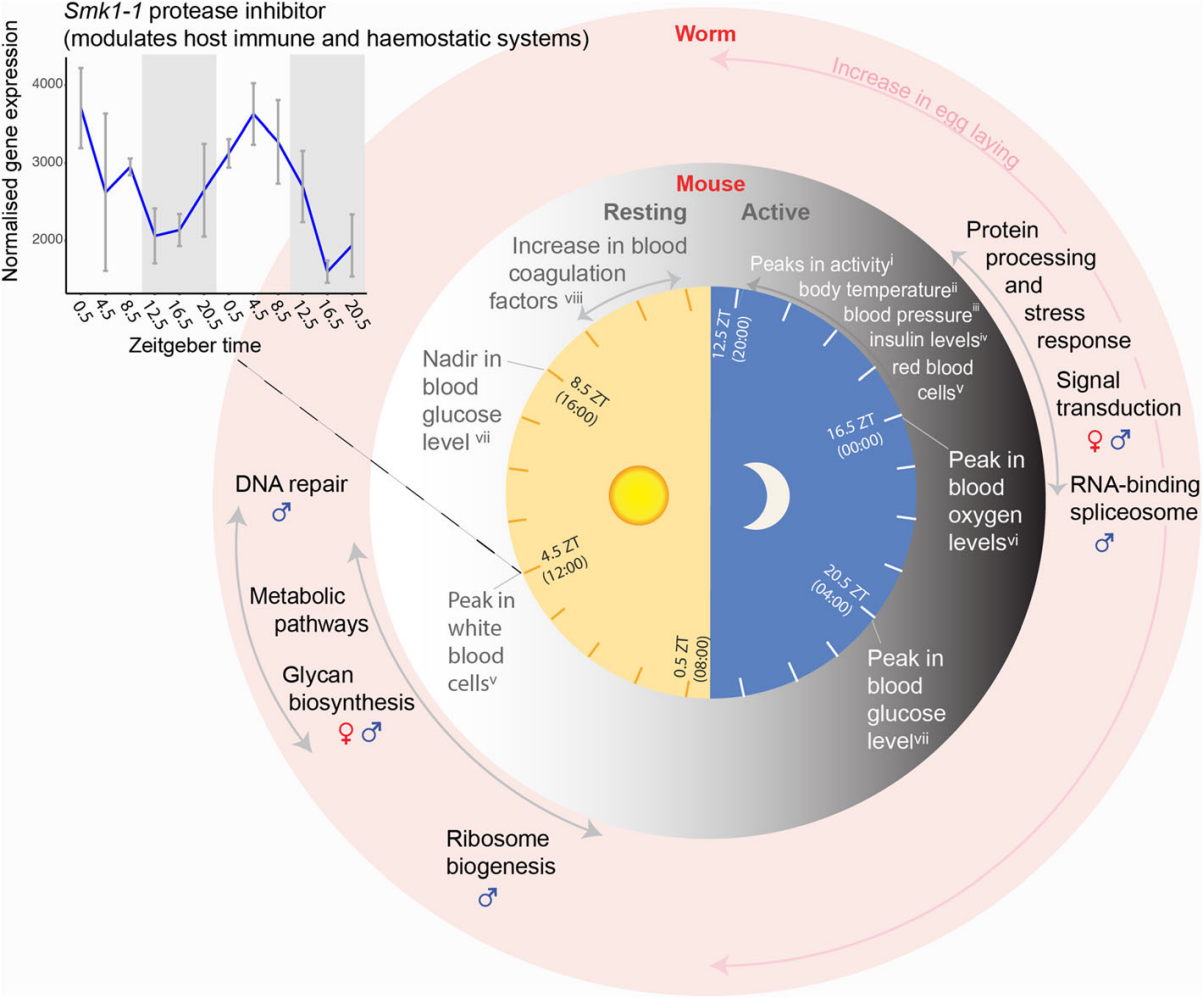
Schematic summarising daily rhythms in the transcriptomes of adult *Schistosoma mansoni* and the mouse vasculature. Rhythms in the transcriptomes of *S. mansoni* identified from diel gene functional enrichment analyses and rhythms in the mouse vasculature summarised from the literature (^i^[21], ^ii^[15], ^iii^[16], ^iv^[78], ^v^[17], ^vi^[18], ^vii^[19], ^viii^[81]). Side plot showing temporal expression profile of *Smk1-1*(Smp_307450), the diel gene with the highest amplitude in male worms and a proposed vaccine candidate. Exponentiated, VST-normalised gene expression values are shown, with means taken for each time point. Error bars show the standard error of the mean

We found diel genes known to be involved in interactions between *S. mansoni* and its host, and most peaked during the day. These included six diel genes involved in N-glycan and glycosaminoglycan synthesis, and glycosylation [32], five of which peaked during the day (Additional file 1: Table S12), including mannosyltransferase (Smp_150200) that is a marker for a tegumental cell type (Figs. 3 and 4; Additional file 1: Table S10). Three others encode enzymes involved in the synthesis of heparin-like glycosaminoglycans that may increase anti-coagulation activity of mammalian host blood [33]; putative beta-1,3-glucuronyltransferase (heparin-like) (Smp_083130) and Zinc finger CCHC domain-containing protein 4 (Smp_245920) peaked during the day in females, whereas putative heparan sulfate N-deacetylase/N-sulfotransferase (Smp_134250) cycles in males, but peaked at night (Additional file 1: Table S11). Three more genes involved in host-parasite interactions were found cycling in males only and all peaked at midday (ZT 4.5): *SmKI-1* (Smp_307450; Fig. 6; see Additional file 3: Supplementary information 1 [34–36]), *carbonic anhydrase 12* (Smp_168730) and *Venom-allergen-like 12* (*Val12*, Smp_123540). SmKI-1 is a tegument localised protein [35] and the latter two are single cell markers for tegumental cell types (Fig. 3). *Val12* is a marker for late tegumental progenitor cells (Sm13^+^ cells) [25], and we show it expressed in approximately 500 cells that are positioned along the entire length of the male body (Additional file 2: Fig. S7). *Val12* transcripts reach the body wall musculature, with some extending in the tubercles of the dorsal tegument (Fig. 5C, D, Additional file 2: Fig. S7); this supports protein structural data indicating that it is likely to be secreted/excreted, onto the tegument or into the host environment [37].

In males, two additional day-time networks of interacting genes were predicted. The first was formed of four genes involved in DNA repair (Fig. 5E, Additional file 2: Fig. S6), and the GO term ‘damaged DNA binding’ was significantly enriched (FDR = 0.0036, Additional file 1: Table S10; Additional file 2: Fig. S8). The second included genes putatively involved in ribosome biogenesis (Fig. 5F, Additional file 2: Fig. S6), e.g. a putative AAA family ATPase (Smp_160870) which also exhibits diel expression in females and whose human ortholog (NVL) regulates 60S ribosomal subunit biogenesis in the nucleolus in a spatiotemporal manner [38].

The transcriptome patterns associated with the female reproductive system are particularly interesting because schistosome eggs are the cause of disease in the host (including granuloma formation after becoming embedded in the liver and intestine) and transmission. Seventeen of the 21 diel genes that are markers for cell types of the female reproductive system (the vitellarium, germline and Mehlis’ gland cells) peaked during the day, between 2.5 and 6.5 ZT. These included a *Trematode Eggshell Synthesis domain-containing protein* (Smp_326610; Table 1, Fig. 4A, Additional file 1: Table S12) that is a major structural component of the egg shell in schistosomes [39]. Also expressed in phase were other diel genes potentially involved in reproduction (but not identified as cell type markers): a *vasa-like DEAD box ATP-dependent RNA helicase* (Smp_154320, expressed in mature oocytes, *Smvlg2* [40]) and a second *putative eggshell protein* (Smp_340010). To investigate whether transcriptional rhythms might reveal patterns in egg laying, we recorded day and night egg counts for individual worm pairs in vitro. For 54 worm pairs in total, we calculated day and night egg counts as the median of three consecutive days and nights, respectively (three replicates of each). Females, on average, laid 13 more eggs over the 12-h night-time, corresponding to a 50% increase compared with the 12-h day-time: for biological replicate 1, *n* = 12 females/pairs, median difference in night–day egg counts = 12.0, paired Wilcoxon test *p* = 0.003; biological replicate 2, *n* = 17, paired Wilcoxon test *p* = 0.002, median (night-day) = 5.3; replicate 3, *n* = 17, *p* = 0.0003, median (night-day) = 17.5; and replicate 4, *n* = 8, *p* = 0.008, median (night-day) = 14.3 (Fig. 4D; Additional file 2: Fig. S9).

### *Schistosoma mansoni* is missing most metazoan core circadian clock genes

Of the 33 genes that cycle in both sexes, 17 showed identical phases and the remaining ones peaked within 4 h of the opposite sex (Additional file 2: Fig. S10; Additional file 1: Table S7). These mRNA oscillations, in phase between the sexes, suggest external cyclical cues drive the synchrony of these rhythms within the *S. mansoni* pairs (and population) inside the mammalian host. Host rhythms may be driving the parasite rhythms, and doing so either directly or by entraining an endogenous time-keeping machinery within the parasite—an intrinsic circadian clock.

This led us to investigate if *S. mansoni* has homologues of the canonical animal circadian clock genes and whether they show 24-h periodicity in our datasets. Extensive BLASTP searches revealed that core elements of the negative feedback loop appear to be missing. Even when the stringency was relaxed to an *E*-value of 1 (BLAST), there were no putative hits for Period or any Cryptochromes (Additional file 1: Table S13). We found two DNA photolyases (Additional file 1: Table S15), which are members of the cryptochrome/photolyase family (CPF), but they clustered with the CPD photolyases, not the canonical animal circadian-related Cryptochromes (Additional file 2: Fig. S11), and they lacked an expected FAD-binding domain (see Additional file 3: Supplementary information 2 for secondary structural features of putative circadian proteins [41–52]). We identified three bHLH-PAS proteins similar to Clock (Smp_178780 and Smp_168600) and Cycle/Bmal1 (Smp_341950) (Additional file 1: Table S13 & 14); however, phylogenetic analysis showed that they clustered with the closely related non-circadian proteins ARNT, AHR and SIM (Additional file 2: Fig. S11; Additional file 1: Table S15). Therefore, *S. mansoni* appears to lack the core negative feedback genes Period and Cryptochromes, as well as the positive transcription factors Clock and Bmal1/Cycle. However, we did identify an orthologue of Timeout (Tim2; Smp_163340), but not its paralogue Timeless (Tim1; Additional file 2: Fig. S11; Additional file 1: Table S13 & 14). *Timeout* did not cycle in our datasets.

We identified putative homologues for many secondary clock genes, including orthologues for vrille, slmb/lin23, shaggy/GSK3 and doubletime/Ck1e/KIN-20 (Additional file 2: Fig. S12). However, none of the orthologues or other homologues (Additional file 1: Table S13 & 14) has diel expression, even in the male head samples. Although *S. mansoni* orthologues of metazoan clock genes did not show 24-h periodicity, we found 51 diel genes in *S. mansoni* whose orthologue in another animal also has ~ 24-h oscillations in expression (Additional file 1: Table S7 & 16). *S. mansoni* has 24 diel genes in common with *Drosophila melanogaster* (14 genes for male worms, *p* = 0.03927; 15 for females, *p* = 0.000687, Fisher’s test), 32 with mouse (23 for male *p* = 0.5179; 17 for females *p* = 0.6248) and only 4 with another lophotrochozoan, the limpet *Cellana rota* [53] (4 for females *p* = 0.01117; 2 for males *p* = 0.3675).

### Diel genes include potential therapeutic targets

An RNAi screen to uncover new therapeutic targets in *S. mansoni* identified 195 genes that caused parasite detachment and affected survival [54], eight of which we show have 24-h periodicity in transcript expression (Additional file 1: Table S7), including *Hsp90* (Smp_072330) that had very high amplitudes in both sexes. By searching the ChEMBL database, we identified existing drugs that are predicted to target the encoded protein of 26 diel genes, 12 of which are phase IV approved drugs (i.e. with the best safety record for humans), including four metabolic chokepoints (Additional file 1: Table S17). The diel genes with the highest amplitudes in each dataset all have existing drugs predicted to target their products; for example, *SmKI-1* (Smp_307450) has four phase IV compounds that are predicted to target it (Additional file 1: Table S17), and it is also a proposed vaccine candidate [55].

## Discussion

Growing evidence is revealing the importance of biological rhythms in parasites as a strategy to optimise survival within a host and transmission between hosts [1]. Behavioural patterns have been recorded for decades [6 and references therein], but only in the last 5 years have the underlying molecular oscillations that are responsible for these rhythms been investigated in unicellular parasites [7–9]. In this study, we have discovered the first 24-h rhythms in the transcriptomes of a metazoan parasite, revealing that, like free-living animals, the effects of the earth’s daily rotation likely influence the biology of this intravascular flatworm. The number of diel genes is low (< 2% of transcriptome) compared to other animals; e.g. 7.9% of expressed genes in pooled, whole *Daphnia pulex* water fleas [2], and 43% of protein-coding genes from mouse organs [14]. The amplitudes of diel genes in *S. mansoni* were also comparatively low (median fold change of 1.18–1.24 in *S. mansoni* compared to 2 in *D. pulex* [2]). The low number of diel genes in *S. mansoni* could be the result of sampling pooled, whole worms; we may be detecting only a proportion of rhythmic genes, those whose expression phase is the same across tissues within a worm, between worms within a pool (from one mouse), and between pooled worms from the three host mice at each time point. The low amplitude of diel genes may also be due to this technical issue. Diel genes in male head samples had a higher median amplitude than the whole-body samples, suggesting that organ-specific cycling might have been dampened in our whole animal samples. It will be interesting to see the extent to which there are differences in diel expression patterns between tissues, as specific cell types and organs are sampled at this temporal scale, facilitated by recent advances in single cell transcriptomic methods [25, 56, 57]. If future studies reveal phase differences between tissues and organs, this could suggest that peripheral rhythms (and/or clocks) may not be synchronised in a hierarchical manner by a master circadian clock/pacemaker cells in the brain (as some are, in mouse and *Drosophila*, for example [58, 59]). Technical issues aside, *S. mansoni* adults live in the mammalian vasculature, a relatively predictable environment with muted oscillations (e.g. temperature), compared with the external environment. Living in a comparatively low amplitude environment may explain why we see fewer diel genes, and lower amplitudes in those genes, compared with the diel genes of free-living (non-parasitic) animals. However, the number of diel genes, and amplitude, is also low compared with the blood-dwelling protozoan parasite *Plasmodium chabaudi*. Over 80% of the transcriptome in this single-celled organism shows 24-h periodicity, with cycling genes having high amplitudes (median circadian fold change = 6.5, [8, 9]). But, *P. chabaudi* undergoes a complete cycle of development in 24 h, quite unlike what we see in the multicellular, long-lived adult *S. mansoni*.

Unlike other studies of which we are aware, we introduced an initial filtering step in our analysis. We excluded those genes that were not differentially expressed over time, prior to determining whether or not they were cycling. The aim was to reduce false-positive genes that had low amplitude cycling patterns due to chance fluctuations in the data. Additionally, this step reduced false negatives, by reducing the effect of the multiple hypothesis testing correction upon determining which genes were cycling using JTK_Cycle. This approach may therefore be useful in future studies of diel and circadian rhythms in transcriptome data.

Our analyses revealed distinct rhythmic processes up-regulated during the night and day in the transcriptomes of *S. mansoni* adults, and we have interpreted these rhythms within the context of daily oscillations in mouse vasculature (Fig. 6). During the night-time, there was a striking increase in the expression of molecular chaperone genes that are involved in the unfolded protein and stress responses. Molecular chaperones are implicated in a wide variety of cellular processes: stabilising new proteins [60], processing proteins damaged by environmental stressors [61], and cell signalling [62]. Chaperones that have 24-h periodicity in other animals (and are orthologues of *S. mansoni* diel chaperones) are controlled by the circadian clock to regulate protein aggregation and toxicity [63], and maintain the unfolded protein response within a physiologically appropriate range [64]. In *S. mansoni*, an increased unfolded protein response at night might be a reaction to, or anticipation of, periods of high ER-protein-folding demand that could be triggered by host cyclical rhythms, and this may be an adaptive stress response. One possible trigger is the daily body temperature cycle of the mouse, which increases by ~ 2.5 °C as the mouse becomes active at the onset of the dark phase [15]. Although relatively moderate, the increase is enough to activate the mouse’s own heat shock response [65]. The daily body temperature cycles also drive a rhythmic alternative splicing program within the mouse [66]. It is a possibility, therefore, that the increase in the mouse body temperature at night also drives a heat shock response and splicing activity in the worms. Several RNA-binding proteins are involved in splicing regulation in response to heat stress: Ser/Arg-rich splicing factors are known regulators after heat shock [67], some hnrpH genes have a role in an arrest of mRNA splicing following heat shock [68, 69] and Hsp70 is known to reactivate mRNA splicing after heat inactivation [70]. Orthologues of these genes in *S. mansoni* form part of the night-time interaction network in male worms (Fig. 2). As temperature entrains circadian rhythms in in vitro populations of the blood-dwelling unicellular parasite, *Trypanosoma brucei* [7], it may be an important cyclical cue for *S. mansoni* as well. Other environmental stressors known to activate the heat shock pathway include hypoxia and reactive oxygen species [71] and as mouse blood oxygen and glucose levels increase during the dark phase [18, 19], they could also induce the transcription of heat shock proteins in *S. mansoni*. Circumstantial evidence points towards the nocturnal peaks in the expression of heat shock and related genes being involved in proteotoxic stress rather than a period of protein synthesis, because it is synchronous with the mouse’s active phase (and accompanying increases in environmental stressors) and in anti-phase (at the opposite time of day) to rhythmic processes involved in translation regulation and protein synthesis (e.g. ribosome biogenesis). The nightly peaks in *hsp90* and *PPIase*, and *PPIase* expression in gametes, suggest possible rhythms in hormone cell signalling related to reproduction. In other animals, these proteins form part of a heterocomplex that chaperones steroid hormones (Additional file 2: Fig. S5) and they are critical for reproductive success [72].

The most prominent day-time process was a single extended metabolic ‘rush hour’ that started at the beginning of the host resting phase. As feeding can act to gate the initiation of metabolic activities [73], this may indicate a period of nutrient uptake in the worms. *S. mansoni* adults take up glucose and amino acids from the host blood directly across the tegument [74], and some diel metabolic genes are markers of tegumental cell types. Worms also ingest blood cells into the digestive system with females thought to feed continuously, and males intermittently [74], but there was no evidence of daily rhythms in genes involved in blood cell feeding. Insulin signalling, activated by host insulin, plays an important role in the growth, development and fecundity of schistosomes [75] and is known to influence the maturation of schistosome eggs and their movement into the intestine [76, 77]. In mice, blood insulin levels peak at night [78] (Fig. 6). In the worms, throughout the night, transcript abundance of genes involved in the insulin signalling pathway increased, and so did egg laying. It is possible, therefore, that the worms’ increase in egg-laying rates coincides with periods of higher host insulin levels. However, because this egg-laying experiment was carried out in vitro, and therefore, in the absence of host cyclical cues, this pattern could be an endogenous rhythm. Host cues, like insulin, could act as zeitgebers to synchronise the parasite’s rhythms to that of its host.

Interestingly, most diel genes that are known to be involved in *S. mansoni* interactions with its host (and increase within-host survival) peaked during the day. These included proteins that are secreted or localised to the tegument, e.g. *SmKI-1* (Smp_307450) [79], *carbonic anhydrase 12* (Smp_168730) [80] and *Val12* (Smp_123540) [37]. The SmK1-1 protein inhibits host proteases (including neutrophil elastases) involved in triggering the immune response [79] and interferes with host coagulation pathways to delay blood clot formation [35]. *SmK1-1* (Smp_307450) reaches its peak at midday (ZT 4.5) (Fig. 6) a few hours before the daily increase of mouse blood coagulation factors [81] and coinciding with the day-time release of mouse neutrophils into the blood from the bone marrow [82], possibly indicating that its diel expression may be anticipating or responding to these host cues. Taken together, our results indicate that for the worms the host’s active phase (night-time) is a period of stress, increased egg-laying and physical activity, whereas during the host’s resting phase (day-time), the worm’s rhythmic processes include metabolism, interactions with the host immune and haemostatic systems, DNA repair and ribosome biogenesis. From this, we speculate that a number of host daily rhythms may be important zeitgebers to the worm’s rhythms, such as heat shock triggers, immune and coagulation factors and insulin.

A daily program of gene expression clearly exists in *S. mansoni.* The lack of most of the animal core circadian clock genes suggests there is either an unusual oscillatory mechanism or a functional endogenous clock has been lost and the *S. mansoni* rhythms are responding directly to host rhythms. The last common ancestor of bilaterian animals is hypothesised to have had all the core animal circadian clock components—Period, Timeless and Timeout, Clock, Cycle/Bmal1 and Cryptochromes [83]—and combinations of these are present in extant Lophotrochozoa [84–86], the superclade to which flatworms belong [87]. The only core clock gene we found in the *S. mansoni* genome was *Timeout*, suggesting all other core clock genes have been lost or have diverged beyond recognition. We identified *S. mansoni* orthologs of the secondary clock genes, vrille, slmb/lin23, shaggy/GSK3 and doubletime/Ck1e/KIN-20. However, neither *Timeout* nor the secondary clock genes showed 24-h periodicity. This could be a sampling artefact due to sequencing RNA from pooled, whole worm samples as clock gene rhythmicity may be limited to a subset of tissues [88], or the phase of clock gene expression can vary between tissues and even between cells within a tissue [89, 90]. However, even in the male head samples (containing a smaller subset of organs and tissues), there was still no 24-h cycling of any clock gene transcripts. The lack of 24-h periodicity in clock transcripts could, alternatively, be due to a lack of rhythmicity at the transcript level, or they may have non-clock functions. Indeed, the adaptive advantage of a clock in environments with neither light nor very high-amplitude environmental cycles is less obvious [91]. Nevertheless, the intravascular environment does have 24-h rhythmicity, albeit with some cycles being relatively low-amplitude, such as temperature, and others being more pronounced, such as blood pressure and glucose levels. As all organisms studied to date that live in 24-h rhythmic environments have evidence of an endogenous circadian time keeper [92], we predict that *S. mansoni* has one too. If, in future studies, any of these daily rhythms are discovered to be endogenous circadian rhythms, then our findings suggest that the *S. mansoni* clockwork must be quite distinct from that in other animals, and novel endogenous oscillators may be discovered within our list of diel genes.

Despite the profound global impact of schistosomiasis, there is complete reliance on only a single drug (praziquantel) for treatment, and evidence of reduced susceptibility in some schistosome populations [93, 94] raises the spectre of drug resistance rendering current control measures ineffective. Consequently, there is a drive to develop a new generation of therapeutics. Understanding the rhythms of target genes and their products will determine how an organ, or organism, will respond to a drug at a specific time of the day, and the timing of drug delivery could have a large impact on the effectiveness of target activation or inhibition [95]. Our discovery of daily rhythms in the transcriptomes of adult schistosomes indicates that chronobiology could be an important factor in the treatment of schistosomiasis. The discovery that some diel genes are drug targets and a vaccine candidate suggests that timing of therapeutic delivery could affect its efficiency. Even non-diel genes may have 24-h rhythms at the protein level. We therefore suggest that future potential drug targets and vaccine candidates should be evaluated over a 24-h period to determine if therapeutic efficiency can be optimised. Although we have described daily rhythms in schistosomes collected from nocturnally active mice, we can assume that some of these rhythms will be inverted in worms infecting diurnal humans. The development of new therapeutics against schistosomiasis should include chronobiological information from the parasite and host wherever possible, and investigating further the temporal periods of parasite vulnerability (e.g. the stress response) and metabolic chokepoint activity (during the metabolic rush hour) holds promise for improving human health.

## Conclusions

This study has advanced our understanding of daily molecular oscillations in organisms to now include a metazoan parasite. Schistosome adults live in the bloodstream of a mammalian host, which is a 24-h rhythmic environment. Our finding that *S. mansoni* adults have daily rhythms in their transcriptomes is, therefore, not entirely unexpected. These daily rhythms in the parasite may be driven by host rhythms, either directly, and/or generated by an intrinsic circadian clock that is entrained to host cues. What is surprising, however, is that exploration of the genome revealed a lack of core clock genes that are generally conserved across other animals, and this is suggestive of an unusual oscillatory mechanism or loss of a functional endogenous clock. Most importantly, our identification of diel genes and daily processes has revealed fine-scale temporal partitioning of biological processes, some of which may serve the particular time-of-day challenges of life within the host, such as the activity of the host immune and haemostatic systems. These rhythmic genes and processes give us insight into how these parasites can survive for decades in this environment, and highlight the need to incorporate daily oscillations in transcript abundance into functional genomic studies aimed at developing and delivering novel therapeutics against schistosomiasis.

## Methods

### Animal procedures

The life cycle of *Schistosoma mansoni* NMRI (Puerto Rican) strain is maintained at the Wellcome Sanger Institute (WSI) by breeding and infecting susceptible *Biomphalaria glabrata* snails and mice. Female Balb/c mice were bred at the WSI and maintained on individual air handling units at 19 to 23 °C and 45–65% humidity. Animals were given access to food and water ad libitum, maintained on a 12-h light/dark cycle and housed in groups of no more than 5 adults per cage. Welfare assessments are carried out daily, and abnormal signs of behaviour or clinical signs of concern are reported. All personnel at the WSI performing welfare checks on animals are trained and assessed as competent by a NTCO (Named training competency officer).

Thirty-six 6-week-old females were percutaneously infected with 200 mixed-sex *Schistosoma mansoni* cercariae collected from 13 infected snails as described [96]. In brief, under isoflurane anaesthesia, the mice were carefully transferred onto individual holders in a bespoke pre-warmed anaesthesia rig and their tails inserted into the test tubes containing the cercariae. After 40-min exposure, animals were removed from the anaesthesia rigs, placed back into their cage and monitored until full recovery from the anaesthesia. For parasite collection (below), mice were euthanised by intraperitoneal injection of 200 μl of 200 mg/ml pentobarbital (Dolethal®) supplemented with 100 U/ml heparin (Sigma-Aldrich), and adult worms recovered by portal perfusion (the portal vein is sectioned followed by intracardiac perfusion with phenol-red-free DMEM, ThermoFisher Scientific, containing 10 U/ml heparin).

### Parasite collection

At 42 days post-infection, groups of three mice were perfused every 4 h for 44 h, and the adult worms collected. The worms sampled in the dark phase were collected from mice euthanised under red light conditions. We collected worm samples 30 min after lights on (zeitgeber time (ZT) 0.5) and then 4 h subsequently giving us collection times of ZT: 0.5, 4.5, 8.5, 12.5, 16.5 and 20.5 over two 24-h periods. ZT: 0.5 corresponds to 8 am in the human 24-h clock, so actual collection times were 08:00, 12:00, 16:00, 20:00, 00:00 and 04:00. At each collection time, worms perfused from mice were washed gently in serum-free DMEM media warmed to 37 °C. Mature, paired male and female worms from each mouse were separated from each other by gently pipetting with a glass Pasteur pipette. Six female worms were pooled in an Eppendorf tube, the media removed and replaced with 1 ml of TRIzol, and the same was done for six male worms. The worms were put in Trizol within 60 min of being perfused from the mouse. The worms were left in Trizol for 1 h at room temperature before being stored at −80 °C. A further 10 mature male worms were uncoupled from female worms, pooled from each mouse and fixed and stored in the RNA stabiliser *vivo*PHIX^TM^ (RNAssist Ltd, Cambridge, UK) at 4 °C for the dissection of heads the following week. We used male heads only as they are bigger and easier to dissect than female heads. RNA was extracted from each pool and sequenced (Fig. 1A). From each mouse at each time point, we therefore collected material simultaneously for three time-series datasets: pooled whole females, pooled whole males and pooled male heads, with 36 samples in male and female datasets and 33 samples of male heads (three perfusions had too few worms to collect for head samples: day1_20:00_b, day2_04:00_b, day2_04:00_c). For each dataset, there were three biological replicates at each time point, a biological replicate being a sample of pooled worms from one mouse.

### RNA isolation, library preparation and transcriptome sequencing

RNA was isolated from the pooled whole worm samples in TRIzol reagent according to the manufacturer’s instructions (Life Technologies). For the male head samples, ten additional male worms per mouse per time point were dissected in *vivo*PHIX^TM^ by cutting posterior to the ventral sucker and anterior to the testes. The heads were rinsed in 50% ethanol and pooled in TRIzol and the RNA extracted as for the whole worm samples. RNA quality was assessed using a Pico RNA kit for the BioAnalyzer (Agilent). Good quality RNA was extracted from all but one sample (male head day2_20:00_c). Total RNA was enriched for mRNA using poly(A) pulldown. The sequencing libraries were prepared using the NEB Ultra II RNA custom kit on an Agilent Bravo WS automation system. All samples had 14 cycles of PCR, which was set up using Kapa HiFi Hot start mix and Eurofins dual indexed tag barcodes on Agilent Bravo WS automation system. Paired-end 75-nucleotide RNA sequencing reads were produced from the pooled worm libraries across six lanes of an Illumina HiSeq2500 v4 sequencing platform. All sequencing data are available through ENA study accession number ERP108923 [97].

### Identification of diel cycling transcripts

Read quality was assessed using the FASTQC quality control tool. Raw reads were mapped to the *S. mansoni* genome (version 7 downloaded from WormBase ParaSite release 14 [98]) using STAR [99]. Read counts were normalised using DESeq2 v1.22.2 [100] with default parameters and the variance stabilising transformation. Principal component analysis was then used to exclude outlier samples by comparing them to replicates. One male sample (day1_12:00_a), two male head samples (day1_20:00_b; day2_20:00_a) and no female samples were excluded (Additional file 2: fig S13).

The popular JTK_Cycle method for identifying cycling genes does not determine whether a transcript varies significantly over time; it only determines whether it follows a pattern similar to a sine wave. So to identify robustly cycling diel genes, we first excluded genes that were not differentially expressed across the time course using the general linear model (GLM) approach in edgeR v3.24.3 [22]. Only genes with Counts Per Million (CPM) > 3 across at least three samples were included in the analysis. In the model design, replicates for equivalent zeitgeber times in each of the two 24-h periods were considered as the same time point. Genes with a *FDR* greater than 0.05 were then excluded. Then to identify genes with ~ 24-h periodicity from those that are differentially expressed over 24 h, we used JTK _cycle [23], using the meta2d function from the MetaCycle software [24]. Default parameters were used, i.e. minimum period 20 h, maximum period 28 h. Genes with a *FDR* < 0.01(JTK BH.Q < 0.01) were identified as cycling. We chose this strict threshold to minimise false positives.

Filtering the genes using edgeR increased the number of genes detected as cycling (i.e. it reduced false negatives); it reduced the input number of genes for JTK_cycle, thereby reducing the effect of the multiple hypothesis testing correction. Without initial edgeR filtering, JTK_cycle and Meta2d (another algorithm in MetaCycle) (BH.Q < 0.01) only identified 15 and 35 diel genes in female worms, 40 and 97 in males and 4 and 5 in male heads (for each algorithm respectively). The combined number of diel genes from the three datasets without edgeR filtering was 58 (JTK_Cycle) and 133 (Meta2d) diel genes (0.45 and 1.2% of the *S. mansoni* (v7) gene set, Additional file 1: Table S1-3) compared to 209 diel genes (JTK_Cycle) (2.1% of genes) after edgeR filtering. These analyses have given us a list of genes that cycle with robust ~ 24-h periodicity and with high enough amplitude to be detected in pooled, whole worm samples.

For visualisation of cycling gene expression, normalised counts for replicates were averaged and then log-transformed to generate heatmaps using the *pheatmap* package with the option scale = ‘row’. The fold change of gene expression over the time points was calculated as the max (peak)/min (trough), taking the mean of the replicates.

### Putative function of diel genes

#### Gene Ontology enrichment analysis of cycling genes

To better understand the function of genes identified as cycling, we performed GO enrichment analysis of our gene lists using topGO [101], with *FDR* < 0.05, node_size = 5, method = ‘weight01’ and statistic = ‘Fisher’. GO terms for *S. mansoni* were downloaded from WormBase ParaSite using BioMart [98] on the 5th March 2020.

#### KEGG pathway mapping

Mapping of *S. mansoni* gene products to the KEGG pathway database was performed on the KAAS server (https://www.genome.jp/kegg/kaas/) using the GHOSTX program and BBH method. The significance of cycling gene enrichment in pathways was assessed using Fisher’s exact test, and resulting *p* values were adjusted using the Benjamini-Hochberg procedure, where maps in the KEGG categories 1–4 (https://www.genome.jp/kegg/pathway.html) were tested. Pathways with *FDR* < 0.05 were considered as significant. For visualisation, the R package Pathview [102] was used.

#### Molecular interaction analysis

Molecular interactions were predicted using the online search tool STRING (www.string-db.org; V 11) [103]. Protein sequences for diel genes were collected from WormBase ParaSite. For day-time peaking genes in the male dataset, the protein sequences were entered as a multiple protein search. Default settings were used to predict interactions with a minimum interaction (confidence) score of 0.4, corresponding to a medium level of confidence. Three further identical analyses were carried out, one for day-time peaking genes in female and one each for night-time peaking genes in male and female worms.

#### Single cell data analysis

We used publicly available single cell transcriptome data from mature adult male and female worms [25] to determine if cycling transcripts were specific to a certain cell type (i.e. cell type markers), specific to a category of cell types (e.g. muscle, neurons, germline, tegument lineage, etc.) or more broadly expressed and found in more than one category of cell type. Processed single-cell RNA-seq data were provided by the authors (R object Whole_Integrate_rmv27_50_RN.rds). We used the hypergeometric test to determine whether each cell type cluster contained more marker genes called cycling in our datasets than expected by chance. This was done separately for each of the male, female and male head datasets. The resulting *p* values were corrected using the Benjamini-Hochberg method. A Python script implementing this method is available from our GitHub page (https://github.com/adamjamesreid/schistosoma_daily_rhythms/).

Seurat uniform manifold approximation and projection (UMAP) plots were used to explore the expression of cycling transcripts across cell types and whether they were ubiquitously expressed, or enriched/specific to one or more cell type [104]. Some of the cycling transcripts identified as cell type markers and enriched in specific cell types were validated by in situ hybridisation (below).

#### Fluorescent in situ hybridisation, immunofluorescence and imaging

We carried out fluorescent in situ hybridisation on two diel genes; Smp_004780 (*PPIase*) and Smp_123540 (*Val12*)*.* Probes, buffers and hairpins for third-generation in situ hybridisation chain reaction (HCR) experiments were purchased from Molecular Instruments (Los Angeles, CA, USA). Molecular Instruments designed probes against Smp_004780 for *PPIase* (Lot:PRA764) and Smp_123540 for *Val12* (Lot: PRA767) from sequences on WormBase ParaSite (WBPS version 14, WS271). The temporal expression profiles of both genes were checked (Additional file 2: fig S14 A & B), and even though *PPIase* peaks at night, its expression was high enough to fix worms during the day. Worms were perfused from mice at 10 am and mature worms were fixed by midday using the following fixation protocol (adapted from [105]): anaesthesia in 0.5% solution of ethyl 3-aminobenzoate methanesulfonate (Sigma-Aldrich, St. Louis, MO) for 15 min to separate male and female worms, incubation in 0.6 M MgCl2 for 1 min, and then into 4% formaldehyde in PBSTx (1xPBS + 0.3% TritonX) for 4 h at room temperature. The worms were rinsed 3 × 5 min in PBSTx and incubated in 5μg/ml Proteinase K (Invitrogen) in 1x PBSTx for 30 min at 37 °C. They were post-fixed in 4% formaldehyde in PBSTx for 10 min at room temperature then rinsed in PBSTx for 10 min. From this point on, the experiments followed the protocol described by Choi et al. [106, 107] and developed for wholemount nematode larvae. We subsequently immunolabelled the *Val12* ISH wholemounts with anti-acetylated tubulin and counterstained with phalloidin by blocking (5% horse serum, 0.45% fish elastin, 0.3% Triton X-100, 0.05% Tween 20 in 5 X SSCT) for an hour at room temperature, incubating in blocking media plus 1:500 anti-acetylated tubulin over night at 4 °C, rinsing in 3 × 30 min in SSCT, adding secondary antibody (1:500) and phalloidin (1:500) in blocking media over night at 4 °C, then rinsing 3 × 30 min in SSCT at room temperature. Samples were mounted using DAPI fluoromount-G (Southern Biotech) and imaged on a confocal laser microscope (Leica Sp8).

#### In vitro egg laying assay

Worms were perfused from one mouse, and twelve pairs of adult worms (still coupled) were placed into individual wells of a 12-well plate containing 3 ml of ABC169 media [108] and kept at 37 °C, 5%CO_2_ in the dark. At 8 am and 8 pm every day for 72 h, eggs were collected from each well and counted, giving 3 day-time counts and 3 night-time counts per worm couple. To collect the eggs (with minimal exposure to light and to room temperature, possible zeitgebers), the well plate was taken out of the incubator for 10 min, and the eggs were gently swirled into the centre of the well and collected in 1 ml of media using a stereomicroscope with low light settings. One millilitre of fresh media (warmed to 37 °C) was then added to each well and the plate returned to the incubator. The eggs collected in media were then counted manually using a stereo microscope. The first 12-h period post-perfusion was discounted to allow the worms to acclimate to the in vitro conditions. This experiment was replicated 3 times, each time with worms freshly perfused from one mouse. The median egg number for each worm for day-time and night-time was calculated, and a paired Wilcoxon test was carried out to determine if there was a significant difference in the number of eggs laid between day or night.

### Identification of core, and secondary, circadian clock genes in *Schistosoma mansoni*

We identified putative *S. mansoni* homologues of animal circadian clock genes using two methodologies. The first was a BLASTP sequence similarity search with a cutoff *e*-value of 1e−10 against the *S. mansoni* genome (v7) in WormBase ParaSite (WBPS release 14), using previously defined circadian protein sequences from UniProtKB [109] and GenBank (Additional file 1: Table S13). Our second method enhanced the robustness of our searches by using respective domains of proteins to identify putative orthologues, as shown before [110]. Briefly, domain identifiers for main clock proteins were selected using Pfam and SMART, and their respective signatures were used to query the BioMart function in WBPS against the entire *S. mansoni* genome (Additional file 1: Table S14). A BLASTP of output sequences in NCBI [111] was used to identify putative homologues of these *S. mansoni* proteins, and all respective hits were aligned in Jalview 2 and illustrated in IBS illustrator [112].

To examine whether hits were orthologous to circadian clock proteins from other animals, phylogenetic analyses on the core clock components were conducted: Timeless/Timeout, Cycle/BMAL1/Arntl and Clock (all are basic helix-loop-helix-PAS proteins) and the Cryptochromes/ Photolyases, and the secondary clock proteins: Vrille, Slmb, Shaggy and Doubletime (for all sequences included in phylogenetic analysis, see Additional file 1: Table S15). Sequences were aligned using CLUSTAL OMEGA [113] and visually examined using Jalview 2 [114]. The aligned sequences were exported into Gblocks 0.91b [115] with allowance for smaller blocks and less strict flanking positions for reduced stringency. Conserved positions (3% for bHLH/PAS, 9% for CDP photolyase, 16% for Timeless) were used to construct a neighbour-joining phylogenetic tree (JTT model) with partial/pairwise deletion and 1000 bootstrap replications in MEGA-X [116].

### Comparison of diel 1-to-1 orthologues in *Schistosoma mansoni* and other Metazoa

We identified 2925 cycling genes in *Drosophila melanogaster* from the Cycling Gene Database (CGDB [117]) and 3233 *S. mansoni*-*D. melanogaster* one-to-one orthologues from WormBase ParaSite [79]. Of the one-to-one orthologues, 420 cycled in *D. melanogaster*, 66 in *S. mansoni* males and 48 in females. For mouse, we identified 9534 cycling genes from CGDB and 2855 one-to-one orthologues with *S. mansoni* using WormBase ParaSite. One thousand one hundred forty-six shared orthologues were cycling in mouse, 57 in male worms and 44 in female worms. To examine common cycling genes between *S. mansoni* and another lophotrochozoan, we used the 221 cycling limpet (*Cellana rota*) transcripts identified by Schnytzer et al. [53]. A total of 38,482 limpet-translated transcript sequences were used with 14,499 sequences from *S. mansoni* (WBPS15) to identify one-to-one orthologues using OrthoFinder [118]. Here we looked for shared orthogroups rather than one-to-one orthologues due to the fragmented nature of the limpet transcriptome assembly. There were 5025 shared orthogroups between limpet and *S. mansoni*. Sixty-seven limpet cyclers and 96 *S. mansoni* cyclers were in shared orthogroups. We used the Fisher exact test to determine whether the number of one-to-one orthologues cycling in both species was greater than expected by chance.

### Identification of drug targets

The *S. mansoni* protein sequences of the cycling genes were used to search the ChEMBL v25 database [119], to identify compounds/drugs predicted to interact with them. We followed the protocol previously described [31] (with some minor differences, as listed in Wang et al. [54]). Briefly, for each *S. mansoni* gene, we identified its top BLASTP hit amongst all ChEMBL targets, as well as any ChEMBL targets having BLAST hits with *E*-values within 1e+5 of the top hit’s *E*-value, and then extracted from ChEMBL the drugs/compounds with bioactivities against those particular ChEMBL targets. To calculate ‘compound scores’ for these drugs/compounds, we prioritised compounds in high clinical development phases, oral/topic administration, crystal structures, properties consistent with oral drugs and lacking toxicity (see [120] for details). The candidate drugs/compounds were filtered by selecting (i) approved drugs (compounds with phase III or IV) that co-appeared in a PDBe [121] (Protein Data Bank in Europe) structure with the ChEMBL target, or had median pCHEMBL > 5, and (ii) selecting medicinal chemistry compounds (with phase II or lower) that had median pCHEMBL > 7. The drugs/compounds were placed into chemical classes, based on ECFP4 fingerprints (see [120] for details). Finally, we filtered the remaining candidate drugs/compounds by (1) checking availability for purchase in ZINC 15 [122] and (2), for each *S. mansoni* target, taking the compound with the highest ‘compound score’ from each chemical class.

## Supplementary Information

**Additional file 1: Supplementary tables 1-17. Table S1.** All genes expressed in female *Schistosoma mansoni* samples over the two 24 hour periods including those with ~ 24 hr rhythmicity. **Table S2.** All genes expressed in male *Schistosoma mansoni* samples over the two 24 hour periods including those with ~ 24 hr rhythmicity. **Table S3.** All genes expressed in male head samples of *Schistosoma mansoni* over the two 24 hour periods including those with ~ 24 hr rhythmicity. **Table S4.** - Genes with differential expression over the two 24 hour periods in female *Schistosoma mansoni* samples including those with ~ 24 hr rhythmicity. **Table S5.** - Genes with differential expression over the two 24 hour periods in male *Schistosoma mansoni* samples including those with ~ 24 hr rhythmicity. **Table S6.** - Genes with differential expression over the two 24 hour periods in male head samples of *Schistosoma mansoni* including those with ~ 24 hr rhythmicity. **Table S7.** - Diel genes from the three datasets (i.e. all genes that are differentially expressed over 24 hours plus cycling with ~ 24 hr periodicity). **Table S8.** - Orthologs of *Schistosoma mansoni* diel genes. **Table S9.** - GO terms enriched in diel genes. **Table S10.** - *Schistosoma mansoni* adult cell types enriched in diel genes. **Table S11.** Diel genes involved in KEGG pathways. **Table S12.** Enrichment of Pfam families, InterPro domains, and KEGG pathways with diel genes. **Table S13.** Putative *Schistosoma mansoni* core and secondary circadian clock proteins. **Table S14.** Domain identifiers for main clock proteins were used to query WormBaseParaSite against the *Schistosoma mansoni* genome to identify putative orthologues. **Table S15.** -Animal circadian clock genes used in BLASTP searches and phylogenetic analyses to identify orthologues in *Schistosoma mansoni.* **Table S16.** One-to-one orthologs that show ~ 24 hr periodicity in *Schistosoma mansoni* and three other Metazoa; *Drosophila melanogaster*, *Mus musculus* and *Cellana rota*. **Table S17.** Diel genes with predicted drug inhibitors.

**Additional file 2: Supplementary Figs. 1-14. Figure S1.** - STRING interaction of night-time peaking genes in female worms*.* **Figure S2. –** Heatmaps of diel genes involved in heat shock response and recovery. **Figure S3-5.** – Diel genes in KEGG pathways ‘Protein processing in endoplasmic reticulum’, ‘PI3K-AKT signaling pathway’ and ‘Estrogen signaling pathway’. **Figure S6.** - STRING interaction of day-time peaking genes in female and male worms. **Figure S7.** – *In situ* hybrisation of *Val12* gene in males. **Figure S8.** – Heatmaps of sex-specific 24-hour rhythmic processes. **Figure S9.** – Day vs night egg counts. **Figure S10.** – Heatmaps of diel genes common to female and male worms. **Figure S11.** – Phylogenies of animal core circadian clock genes. **Figure S12.** – Phylogenies of animal secondary circadian clock genes. **Figure S13.** – PCA plot of all samples collected for male and male head time series. **Figure S14.** – Temporal expression profiles of genes validated by *in situ* hybridisation.

**Additional file 3: Supplementary information 1.** SmKI-1, a Bovine Pancreatic Trypsin Inhibitor/Kunitz protease inhibitor domain protein**. Supplementary information 2.** Clock gene protein structure.

## Data Availability

The three RNA-seq time-series datasets generated and analysed during the current study are available in the European Nucleotide Archive (www.ebi.ac.uk/ena) under the ENA study accession number ERP108923 (https://www.ebi.ac.uk/ena/browser/view/PRJEB26892?show=reads) [97].

## Abbreviations

BH.Q: Benjamini-Hochberg *q*-value
ER: Endoplasmic reticulum
*FDR*: False discovery rate
GO: Gene ontology
JTK: Jonckheere-Terpstra-Kendall
WISH: Whole-mount in situ hybridisation
ZT: Zeitgeber time
KEGG: Kyoto Encyclopedia of Genes and Genomes
HSP: Heat shock protein
*PPIase*: *FKBP-type peptidylprolyl isomerase*
*Val12*: *Venom-allergen-like 12*
WSI: Wellcome Sanger Institute
