# Supplementary material for: Daily rhythms in gene expression of the human parasite Schistosoma mansoni
Source: BMC Biol. 2021 Dec 2;19:255. doi: 10.1186/s12915-021-01189-9 (PMC8638415; doi:10.1186/s12915-021-01189-9)
Supplement: Supplementary file 2 — Additional file 2: Supplementary Figs. 1-14. Figure S1. - STRING interaction of night-time peaking genes in female worms. Figure S2. – Heatmaps of diel genes involved in heat shock response and recovery. Figure S3-5. – Diel genes in KEGG pathways ‘Protein processing in endoplasmic reticulum’, ‘PI3K-AKT signaling pathway’ and ‘Estrogen signaling pathway’. Figure S6. - STRING interaction of day-time peaking genes in female and male worms. Figure S7. – In situ hybrisation of Val12 gene in males. Figure S8. – Heatmaps of sex-specific 24-hour rhythmic processes. Figure S9. – Day vs night egg counts. Figure S10. – Heatmaps of diel genes common to female and male worms. Figure S11. – Phylogenies of animal core circadian clock genes. Figure S12. – Phylogenies of animal secondary circadian clock genes. Figure S13. – PCA plot of all samples collected for male and male head time series. Figure S14. – Temporal expression profiles of genes validated by in situ hybridisation. [file 12915_2021_1189_MOESM2_ESM.docx]

**Daily rhythms in the transcriptomes of the human parasite *Schistosoma mansoni***

Kate A. Rawlinson^1🖂^, Adam J. Reid^1^, Zhigang Lu^1^, Patrick Driguez^1,2^, Anna Wawer^3^, Avril Coghlan^1^, Geetha Sankaranarayanan^1^, Sarah Kay Buddenborg^1^, Carmen Diaz Soria^1^, Catherine McCarthy^1^, Nancy Holroyd^1^, Mandy Sanders^1^, Karl Hoffmann^3^, David Wilcockson^3^, Gabriel Rinaldi^1^, Matt Berriman^1🖂^

1. Wellcome Sanger Institute, Wellcome Genome Campus, Hinxton, UK.
2. King Abdullah University of Science and Technology, Thuwal, Makkah, Saudi Arabia
3. Institute of Biological, Environmental, and Rural Sciences, Aberystwyth University, Aberystwyth, UK

**Corresponding authors**: Kate Rawlinson ([kr16@sanger.ac.uk](mailto:kr16@sanger.ac.uk)) and Matt Berriman (mb4@sanger.ac.uk)

**Legend**

Additional file 2: Supplementary figures 1-14. Figure S1 - STRING interaction of night-time peaking genes in female worms*.* Figure S2 **–** Heatmaps of diel genes involved in heat shock response and recovery. Figure S3-5 – Diel genes in KEGG pathways ‘Protein processing in endoplasmic reticulum’, ‘PI3K-AKT signaling pathway’ and ‘Estrogen signaling pathway’. Figure S6 - STRING interaction of day-time peaking genes in female and male worms. Figure S7 – *In situ* hybrisation of *Val12* gene in males. Figure S8 – Heatmaps of sex-specific 24-hour rhythmic processes. Figure S9 – Day vs night egg counts. Figure S10 – Heatmaps of diel genes common to female and male worms. Figure S11 – Phylogenies of animal core circadian clock genes. Figure S12 – Phylogenies of animal secondary circadian clock genes. Figure S13 – PCA plot of all samples collected for male and male head time series. Figure S14 – Temporal expression profiles of genes validated by *in situ* hybridisation.

**
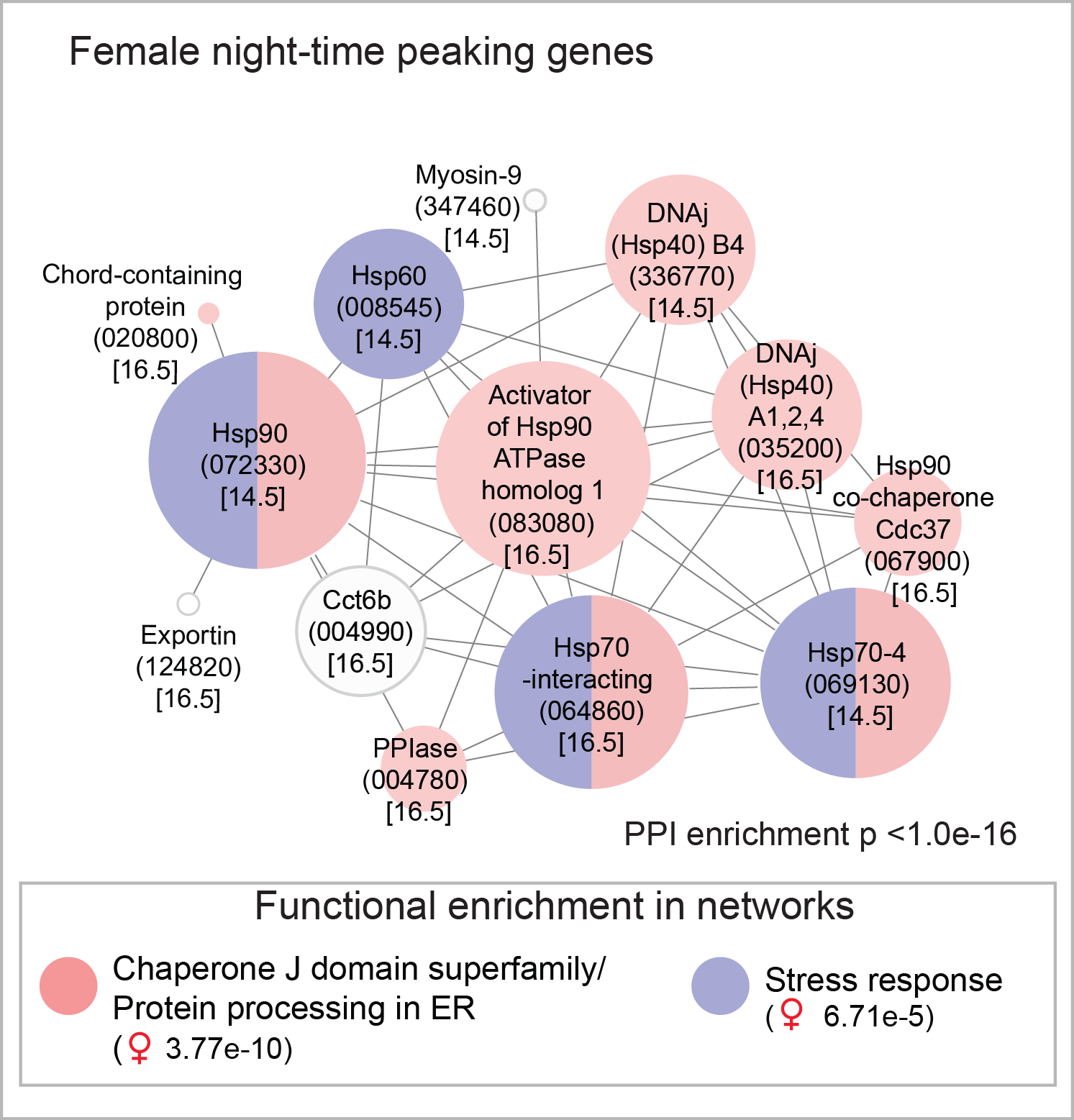
**

**Supplementary figure 1.** Predicted molecular interaction networks of night-time peaking genes in female *Schistosoma mansoni* (computed using the STRING online database). Node size reflects the number of connections a molecule has within the network. Lines (edges) connecting nodes are based on evidence of the function of homologues. Functional enrichment (FDR) as provided by STRING. (PPI= predicted protein interaction; geneIDs with Smp_ prefixes removed; acrophase [time of peak expression; zeitgeber time] in square brackets).


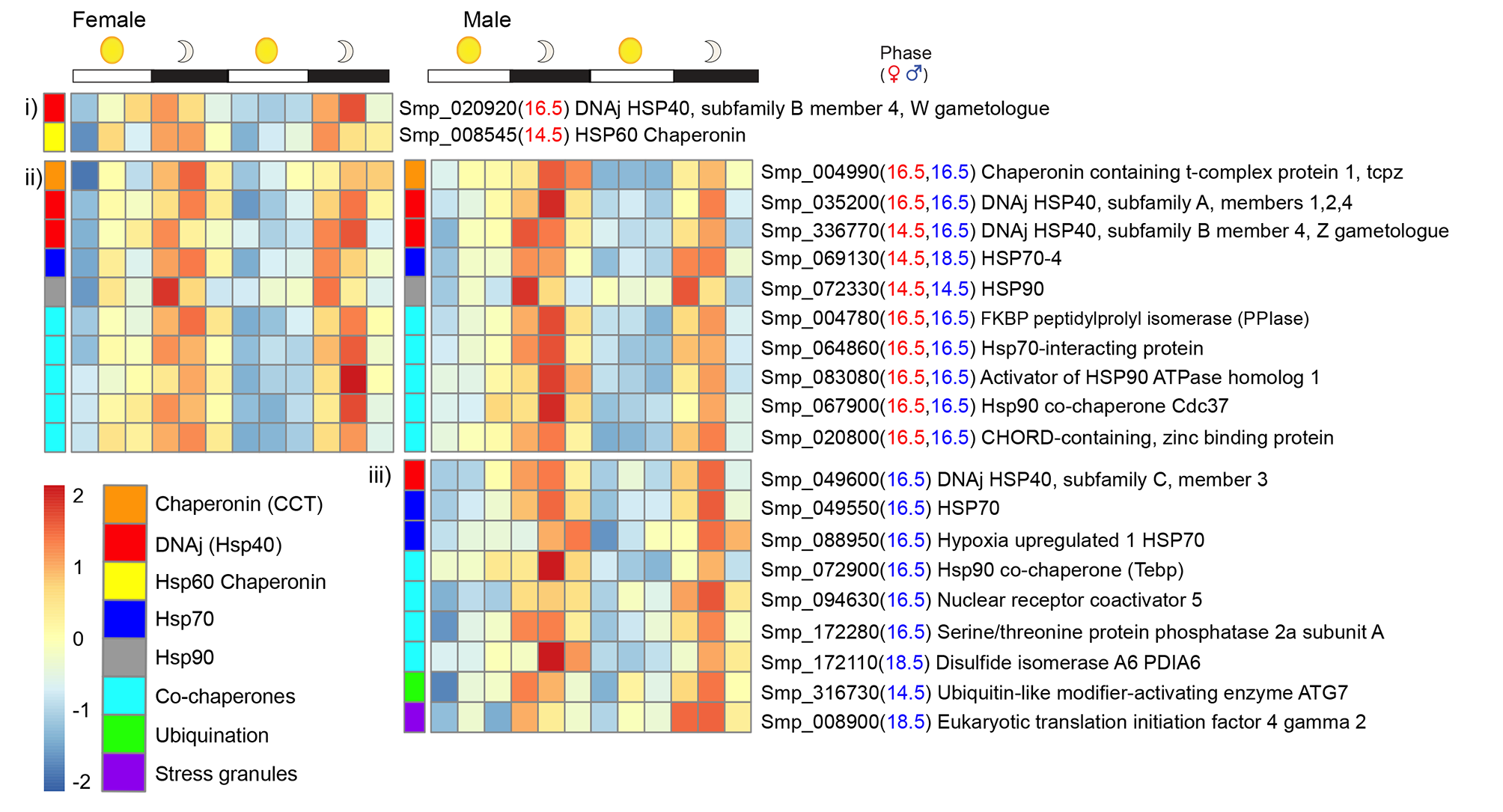


**Supplementary figure 2.** Diel genes encoding heat shock proteins, co-chaperones and other proteins involved in heat shock response and recovery. All reach their acrophase (peak expression) between 14.5-18.5 Zeitgeber time (i.e. 2-6 hours after lights off; 22:00-02:00 local time). Some show diel expression in one sex only (i & iii), whereas another ten cycle in both sexes, with eight in phase (ii).


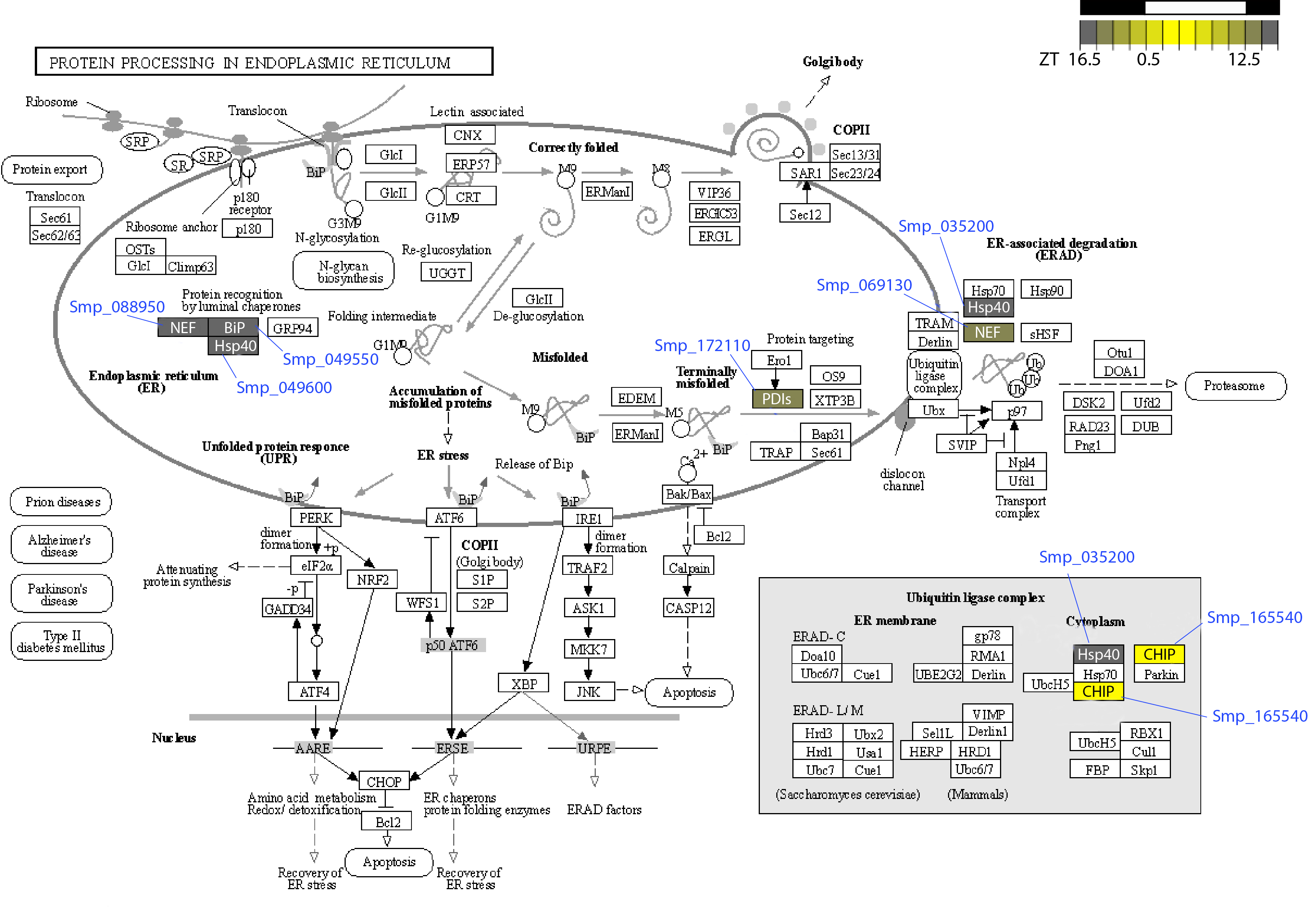


**Supplementary figure 3.** The KEGG pathway ‘Protein processing in endoplasmic reticulum’ includes seven diel genes that encode heat shock proteins and other co-chaperones. Data on KEGG graph rendered by Pathview.


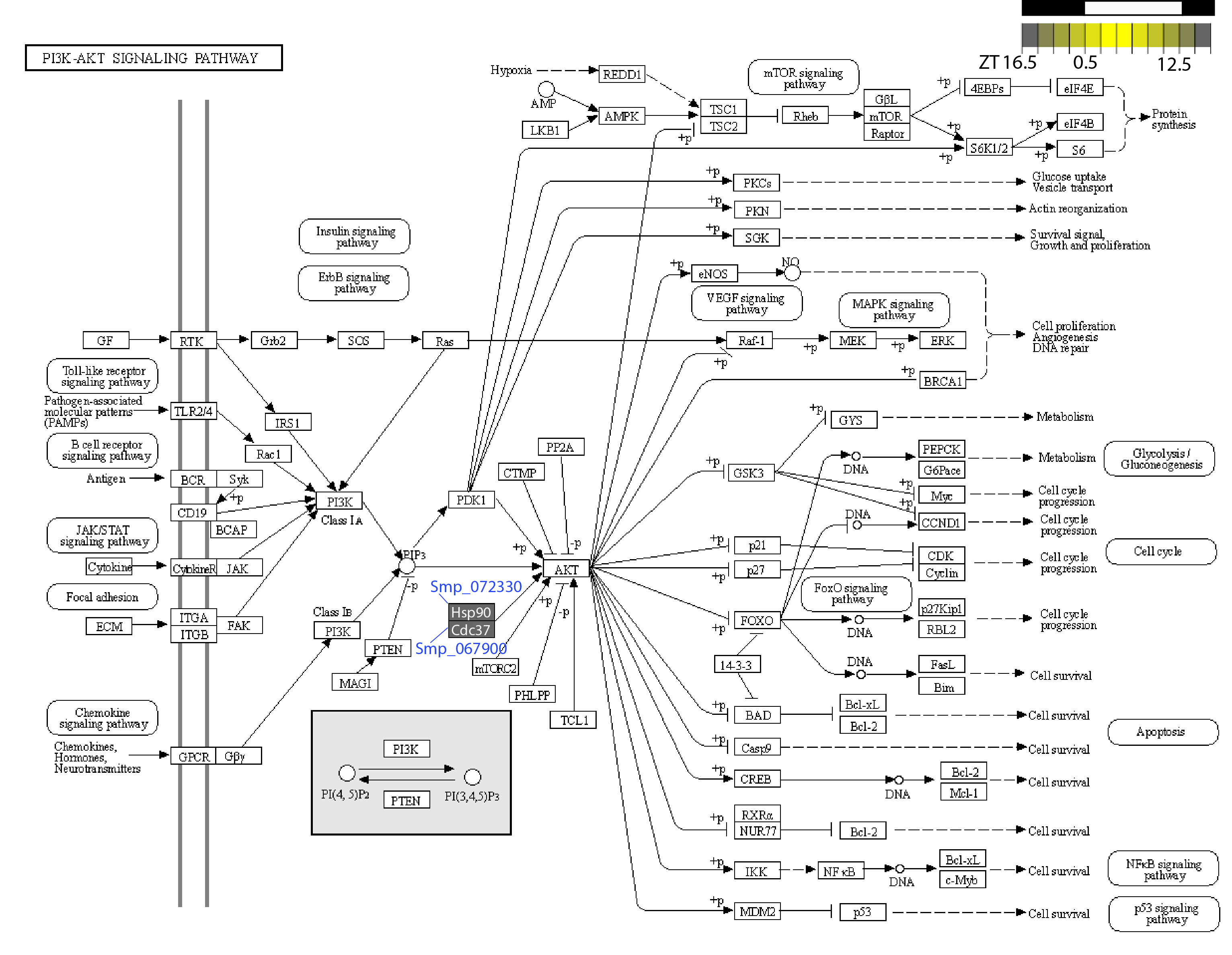


**Supplementary figure 4.** The KEGG pathway ‘PI3K-AKT signaling pathway’ includes two diel genes; one that encodes heat shock protein 90 (HSP90) and the other encodes one of its co-chaperones, cell division cycle 37 (Cdc37). Data on KEGG graph rendered by Pathview.


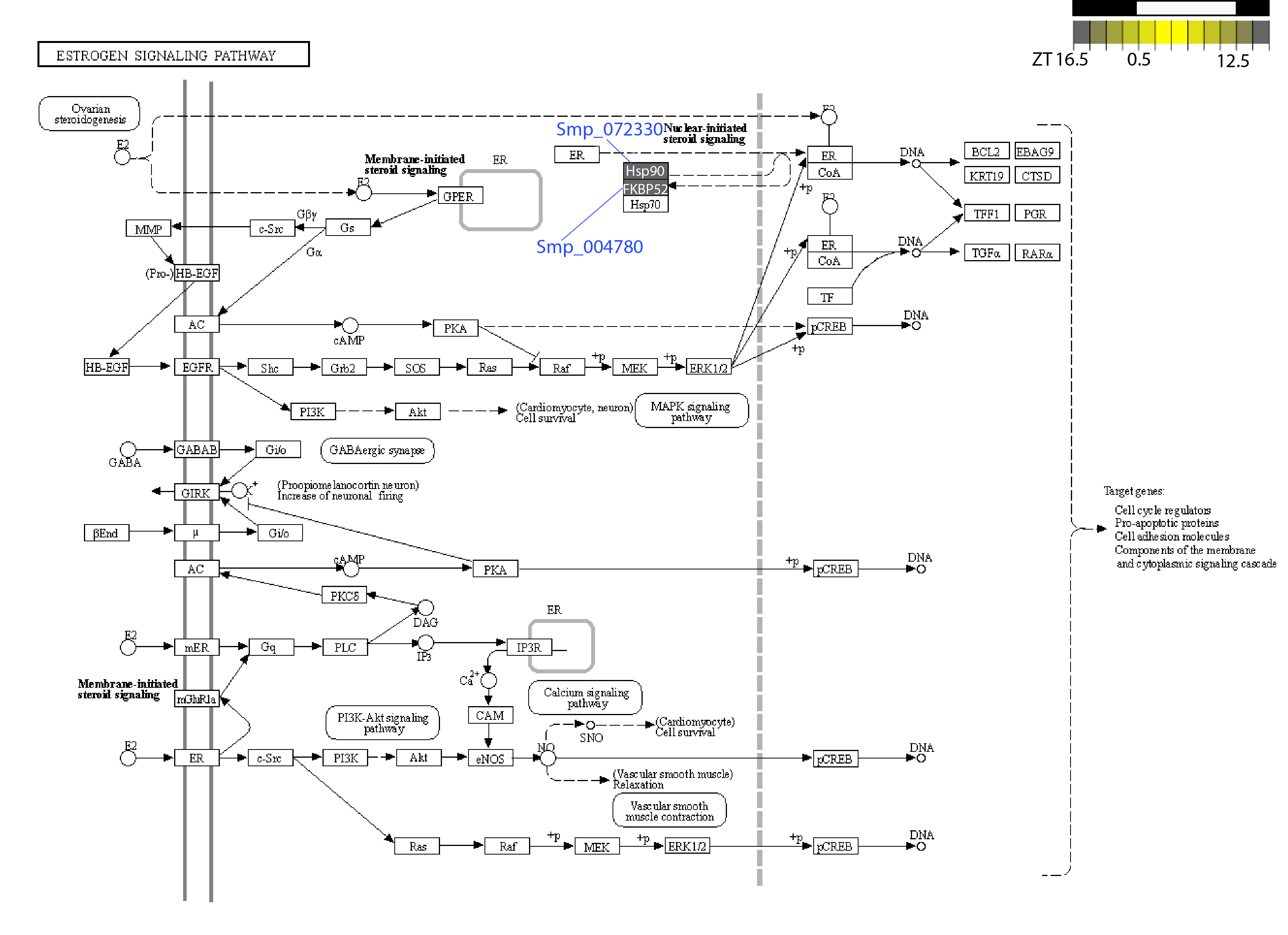


**Supplementary figure 5.** The KEGG pathway ‘Estrogen signaling pathway’ includes two diel genes; one that encodes heat shock protein 90 (HSP90) and the other encodes one of its co-chaperones, FKBP-type peptidylprolyl isomerase (PPIase). However, HSP70 (Smp_303420), another binding partner, does not cycle in male or female worms. Data on KEGG graph rendered by Pathview.


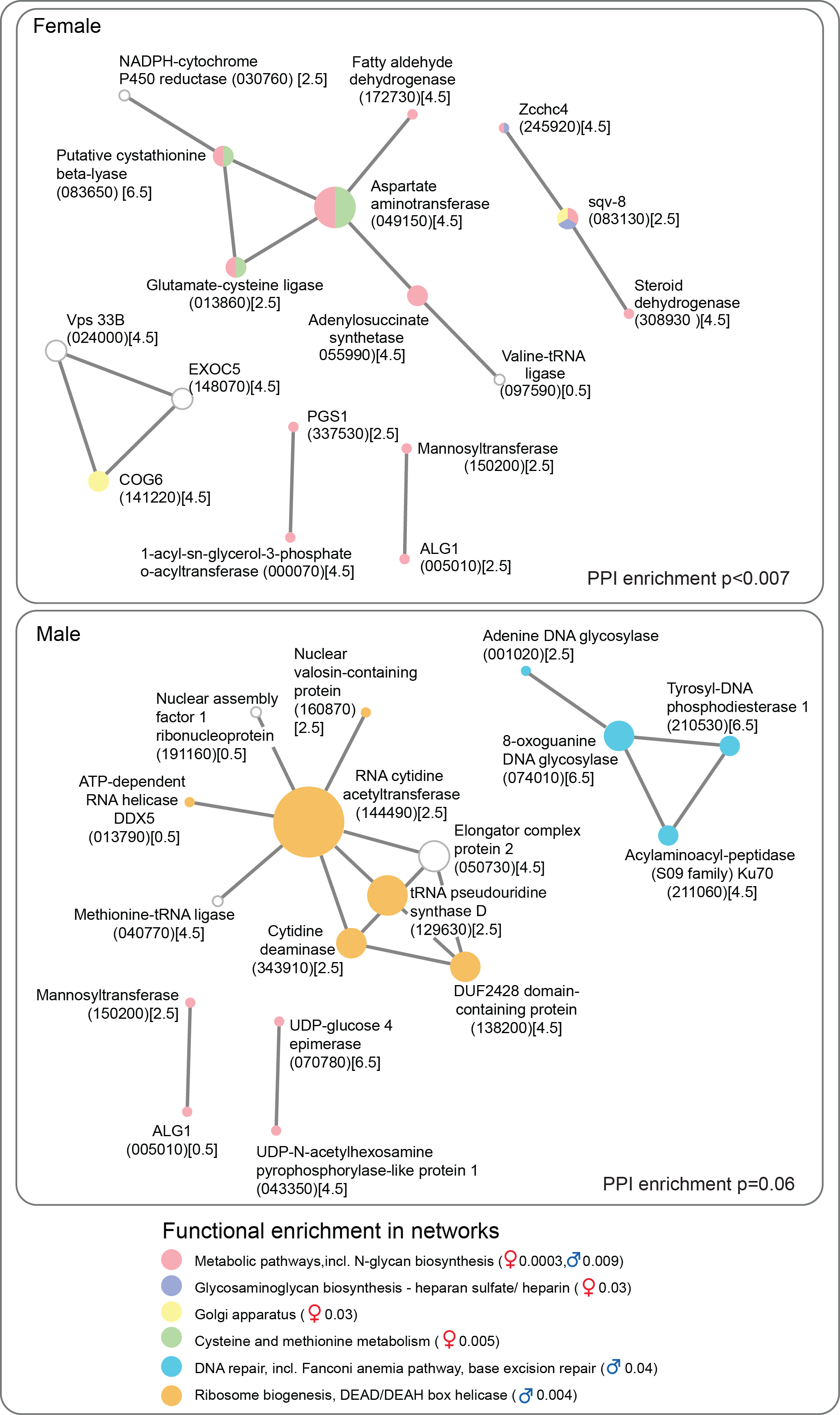


**Supplementary figure 6.** Predicted molecular interaction networks of day-time peaking genes in female and male *Schistosoma mansoni* (computed using the STRING online database). Node size reflects the number of connections a molecule has within the network. Lines (edges) connecting nodes are based on evidence of the function of homologues. Functional enrichment (FDR) as provided by STRING. PPI= predicted protein interaction; “Smp_” prefixes have been removed from gene identifiers for clarity; acrophase/peak expression (zeitgeber time) in square brackets.


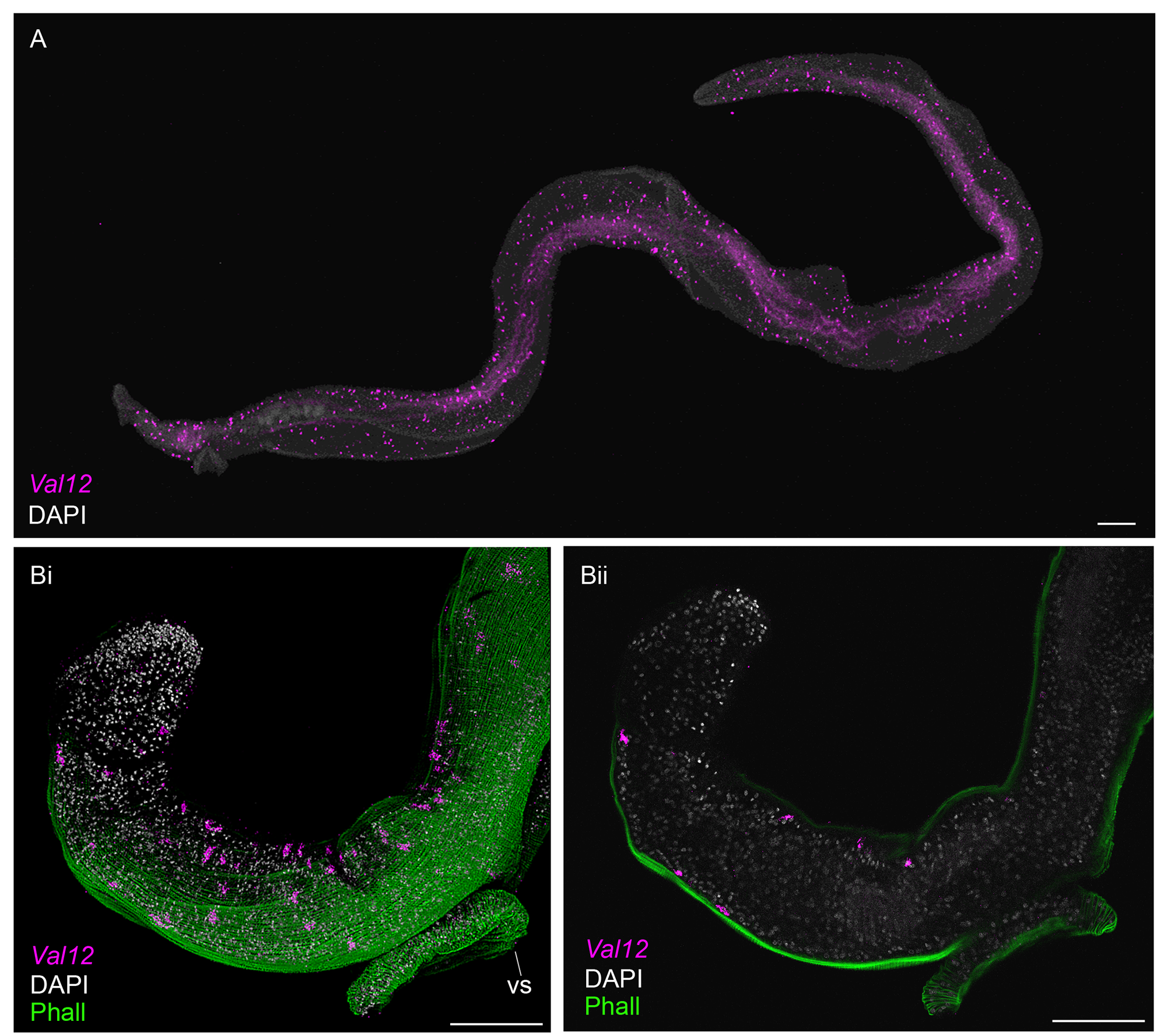


**Supplementary figure 7.** In male worms *Venom allergen-like 12* (*Val12*, Smp_123540) transcripts peak in abundance at midday (ZT 4.5) and are expressed in **A)** ~ 500 cells that are distributed the length of the body (anterior to left)(scale = 200µm). **B)** The *Val12*^+^ cells sit below the body wall musculature (phall = phalloidin) on both the ventral and dorsal sides, **i**) 3D projection and **ii**) optical section of the head (scale = 100µm). vs = ventral sucker, 100% of individuals examined, n = 20.


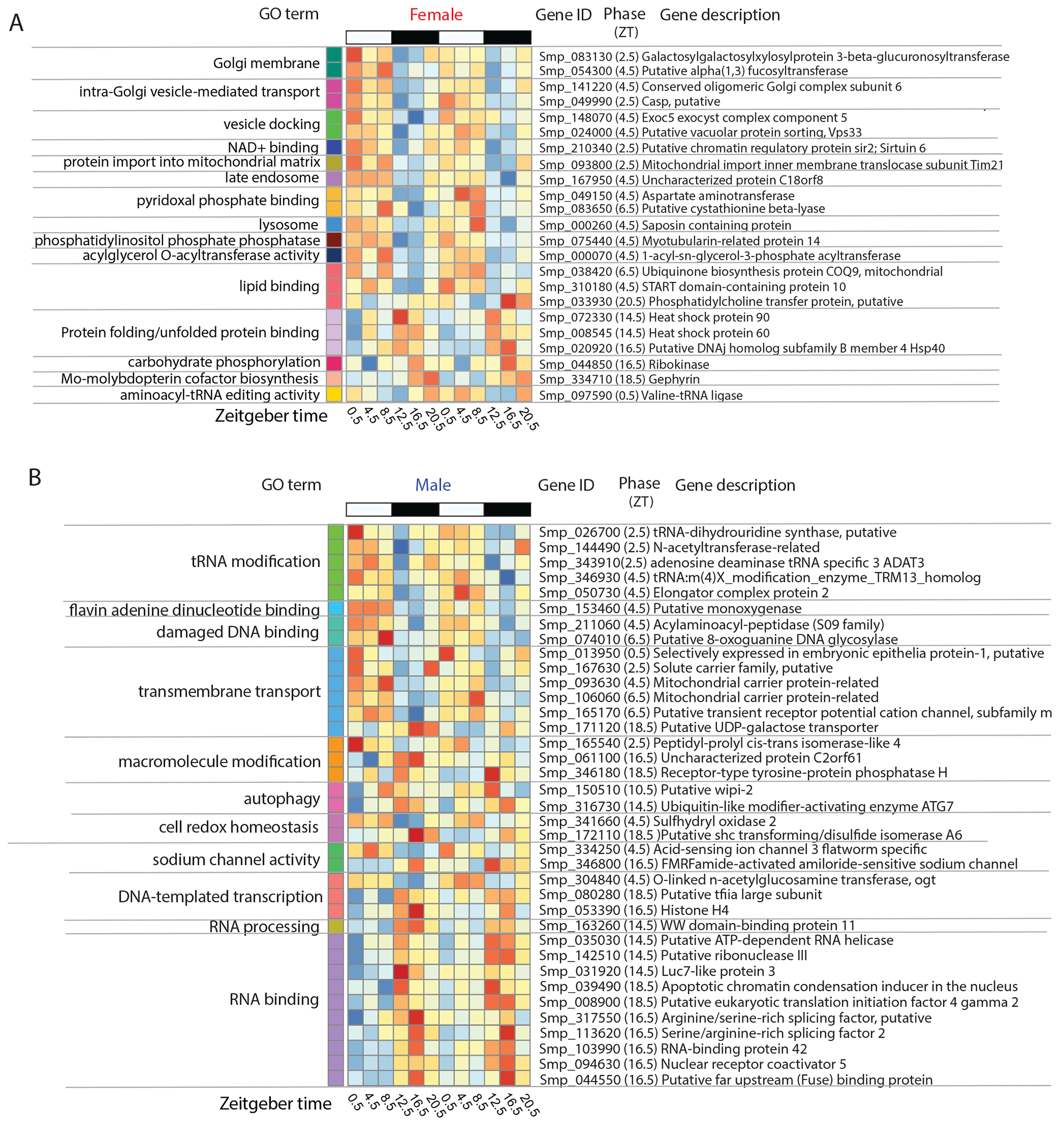


**Supplementary figure 8**. Sex-specific 24-hour rhythmic processes. Heatmaps showing GO terms enriched in diel genes that cycle in females (**A**) or males (**B**) only. (ZT = zeitgeber time).


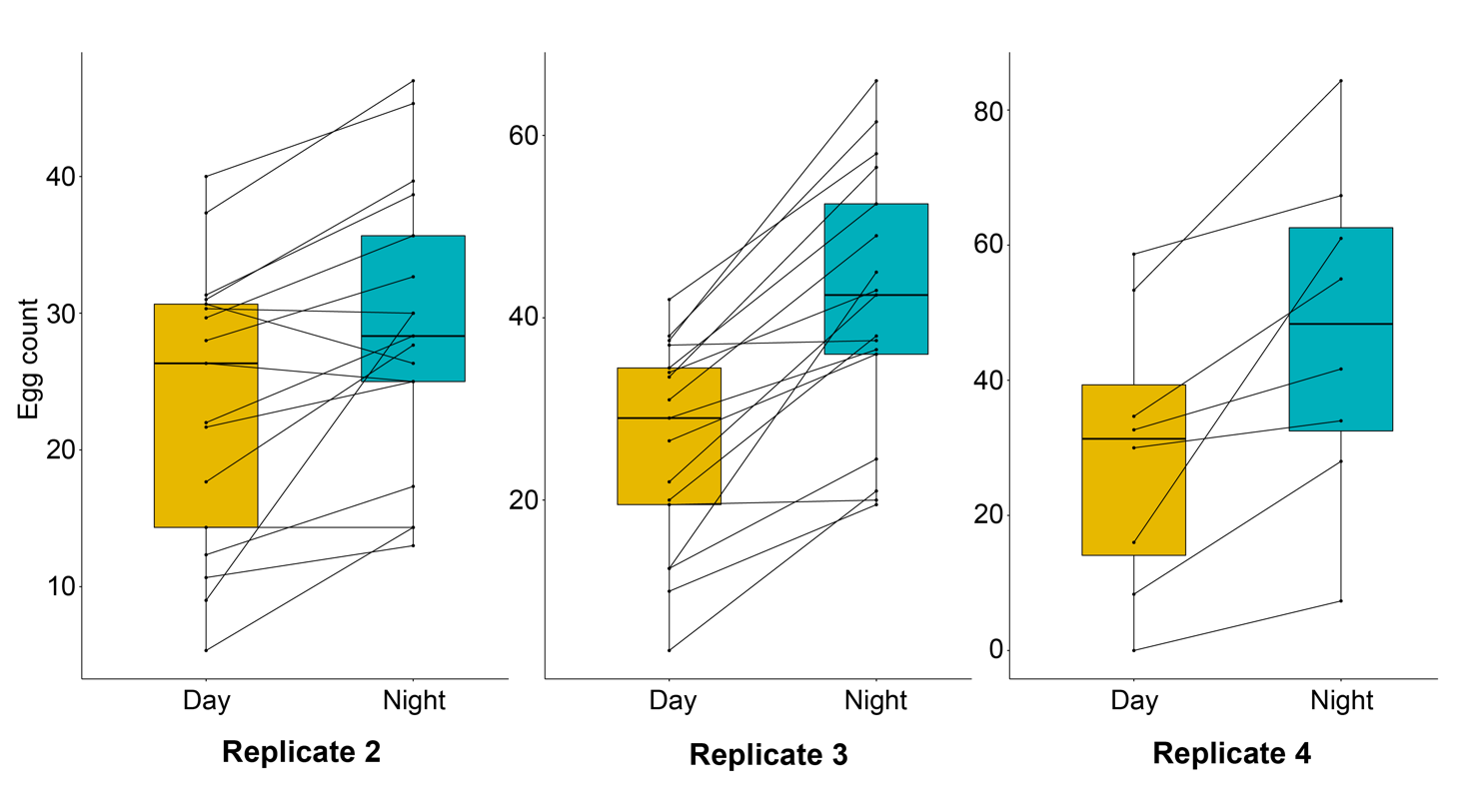


**Supplementary figure 9.** Independent biological replicates of day and night egg counts from paired female worms *in vitro* (median and interquartile ranges)*.* Replicate 2: n=17, paired Wilcoxon test P=0.002, median(night-day)=5.3. Replicate 3: n=17, P=0.0003, median(night-day)=17.5. Replicate 4: n=8, P=0.008, median(night-day)=14.3.


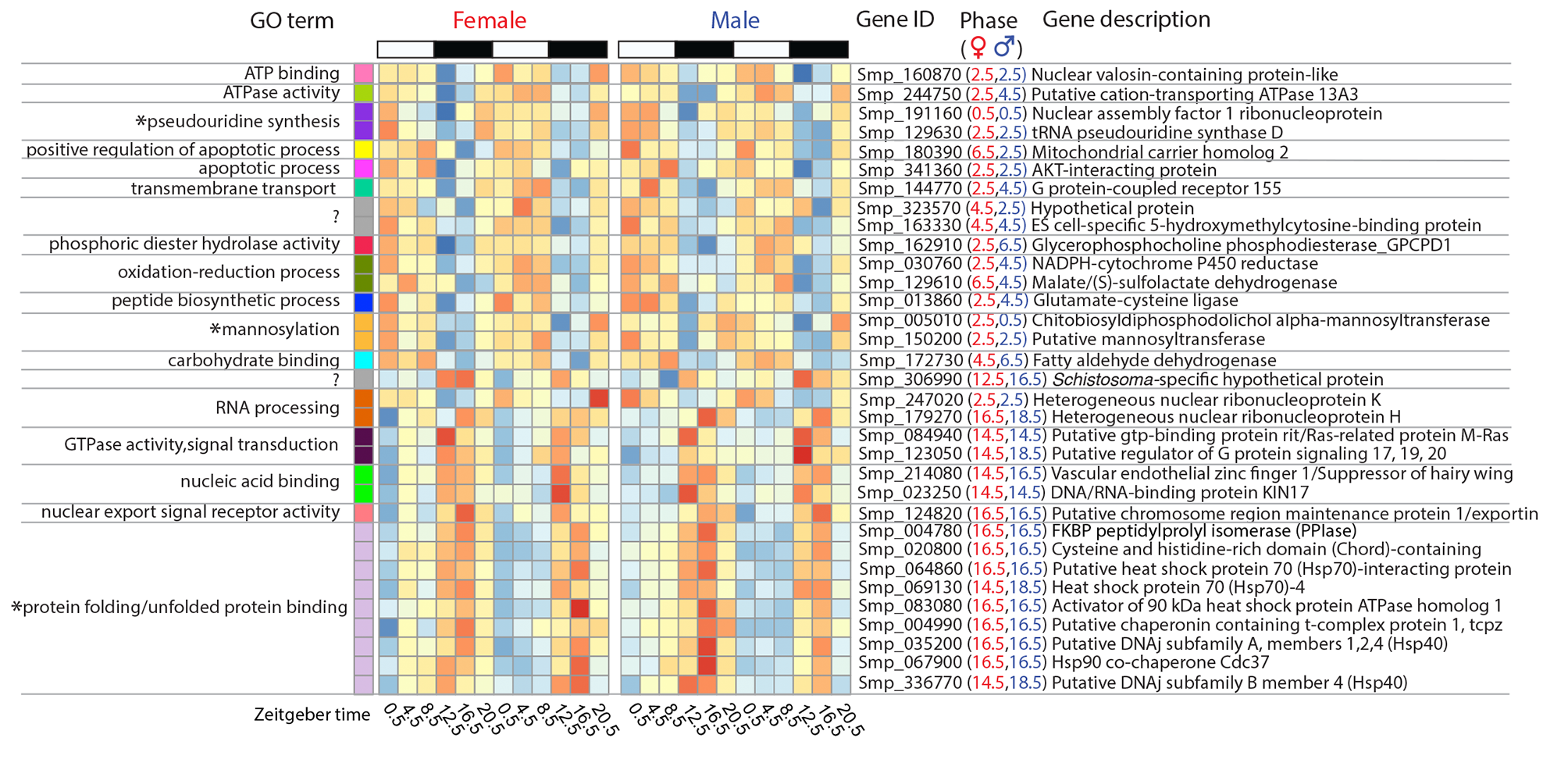


**Supplementary figure 10.** Diel genes common to female and male worms show identical, or similar, phases suggesting that many biological processes and molecular functions are happening in synchrony. Most enriched functions (GO terms) are time-of-day specific; e.g. mannosylation, redox homeostasis and apoptosis occur during the daytime, whereas genes involved in molecular chaperoning, nucleic acid binding and signal transduction reach their acrophase (peak expression given in Zeitgeber time) at night. (* significantly enriched GO terms FDR<0.01, Additional file 1, supplementary table 10).

**
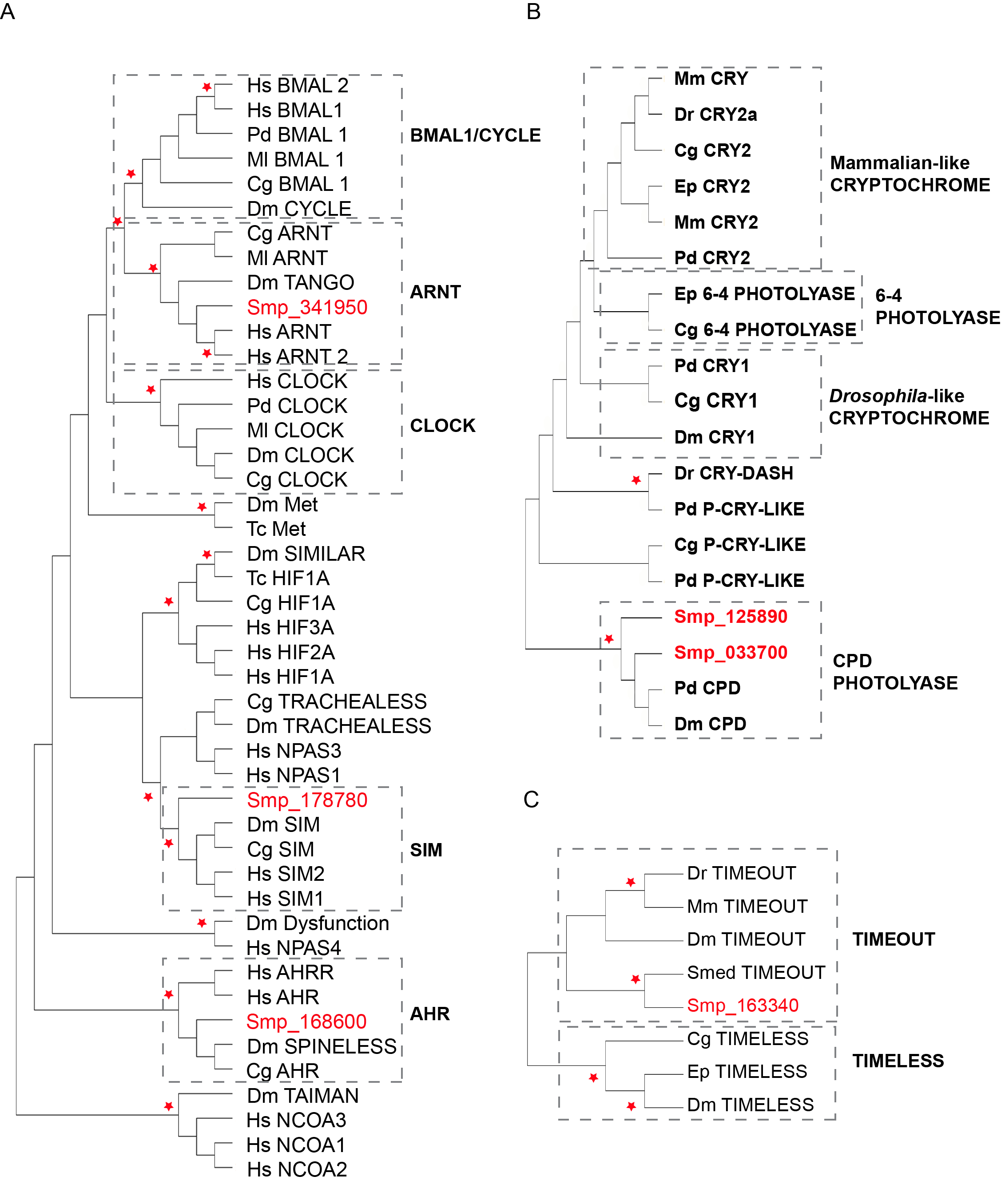
**

**Supplementary figure 11. Core circadian clock gene homologs are missing in *Schistosoma mansoni*.** Neighbour-joining phylogenetic trees were constructed in Mega-X with 1000 bootstrap replications and partial/pairwise deletion (* = bootstrap support >90). **A**) A phylogeny of bHLH/PAS domain proteins shows that *S.mansoni* lacks BMAL1 and CLOCK homologs. Our BLASTP hits cluster with the closely-related non-circadian proteins ARNT, AHR and SIM. **B**) *S. mansoni* has two CPD photolyases but no circadian-related Cryptochromes. **C**) A phylogeny of orthologous genes *tim1* (TIMELESS) and *tim2* (TIMEOUT) shows that our BLASTP hit clusters with *tim2*. We also show that the previously identified *timeless* homolog in the flatworm *Schmidtea mediterreana* [106] clusters with *tim2* homologs of model organisms. Abbreviations: Cg = *Crassostrea gigas*, Dr *= Danio rerio,* Dm = *Drosophila melanogaster,* Ep = *Eurydice pulchra*, Hs = *Homo sapiens,* Ml = *Melibe leonina*, *Mm = Mus musculus,* Pd = *Platynereis dumerilii*, Smed = *Schmidtea mediterranea*, Tc = *Tribolium castaneum*

**
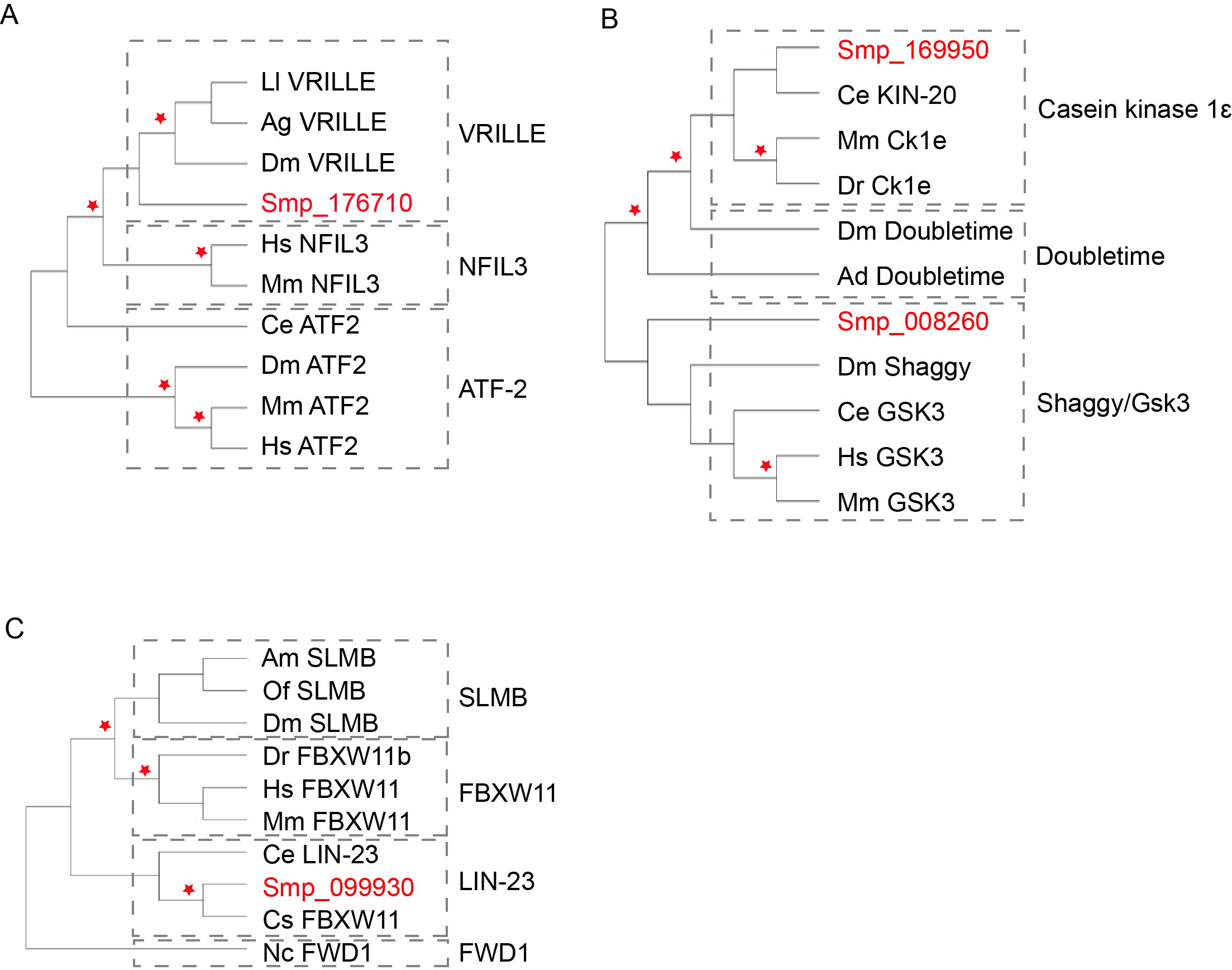
**

**Supplementary figure 12. *Schistosoma mansoni* has homologs of secondary clock genes.** Neighbour-joining phylogenetic trees were constructed in Mega-X with 1000 bootstrap replications and 80% partial deletion using sequences obtained from Uniprot. (* = bootstrap support >90). **A**) Representatives of basic-leucine zipper protein family show insect *vrille* homolog is present in *S. mansoni*. **B**) Smp_169950 clusters with mammalian homologs of *doubletime*, whereas Smp_008260 clusters with *Shaggy*. **C**) Smp_099930 clusters with Lin-23, a previously identified homolog of Slmb in *Caenorhabditis elegans*. Ancestral sequence from *Neurospora Crassa* was used as an out-group. Conserved regions were obtained in Gblocks using least stringency criteria and percentage of all sequences used was as follows: 3% basic-leucine zipper, 11% slmb, 18% shaggy/doubletime. Abbreviations: Ll = *Lutzomyia longipalpis*, Ag = *Anopheles gambiae*, Dm = *Drosophila melanogaster*, Hs = *Homo sapiens*, Mm = *Mus musculus*, Ce = *Caenorhabditis elegans*, Cg = *Crassostrea gigas,* Am = *Apis mellifera*, Of = *Oncopeltus fasciatus* , Dr *= Danio rerio*, Nc = *Neurospora crassa*, Cs = *Clonorchis sinensis*, Ad = *Anopheles darlingi.*

**
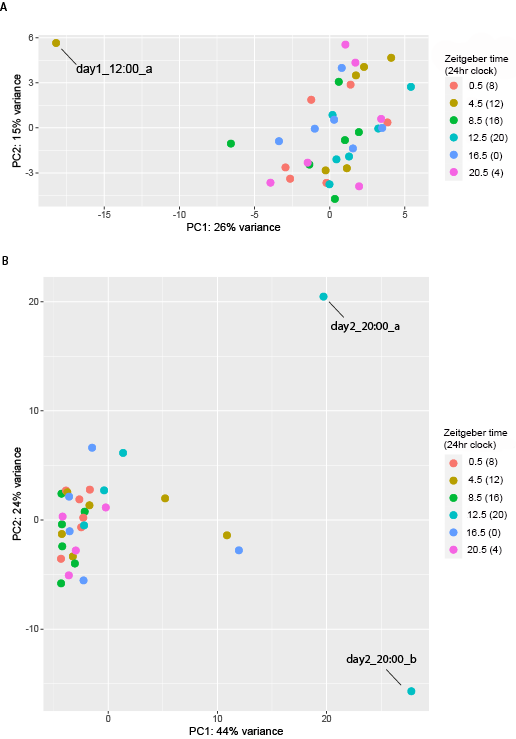
**

**Supplementary figure 13. Principal Components analysis of samples for outlier removal.**

1. Male samples - a single sample (day1_12:00_a) was removed based on distance from all other samples**. B)** Male head samples - two were removed (day2_20:00_a and day2_20:00_b).


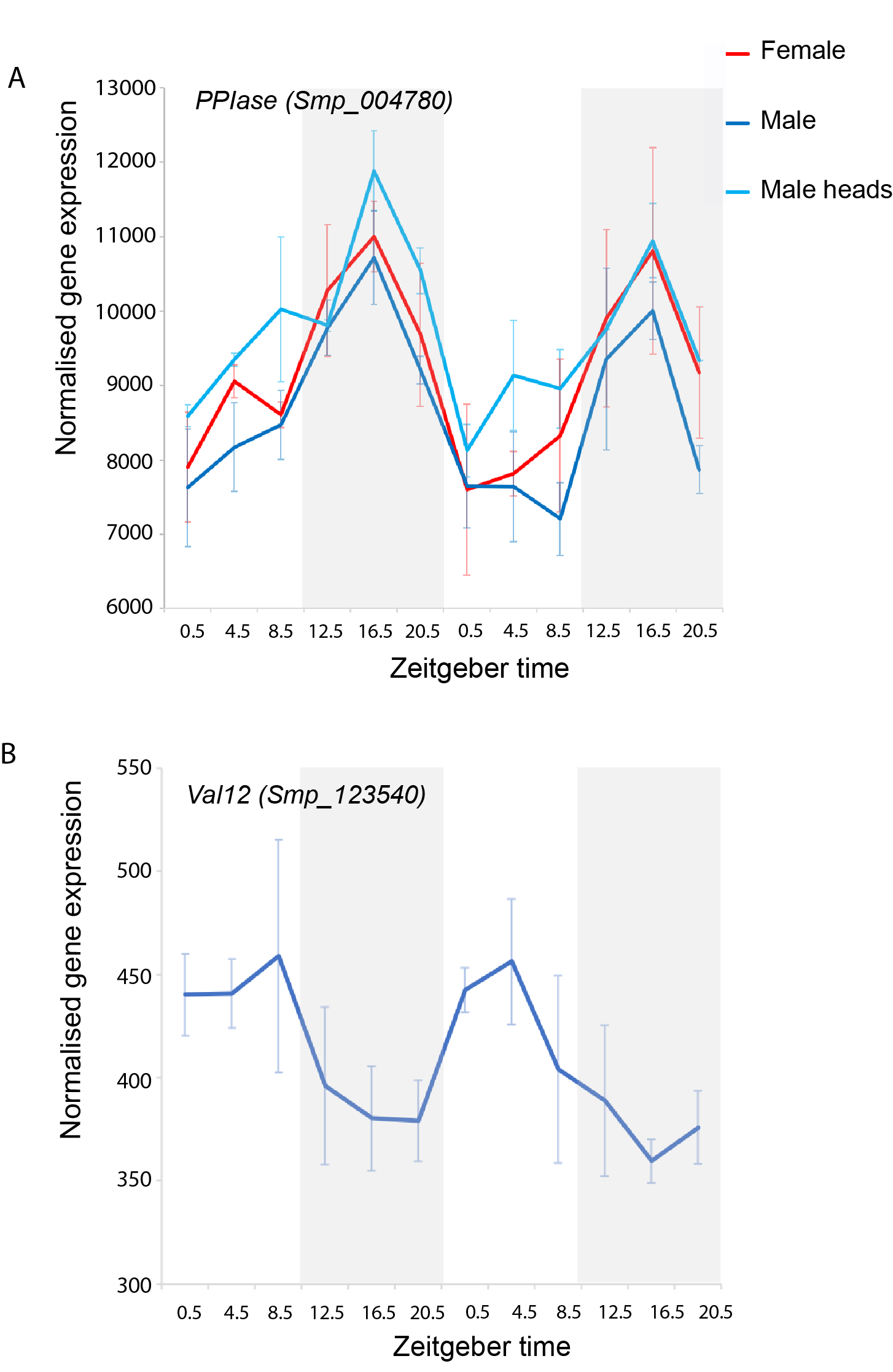


**Supplementary figure 14. Temporal expression profiles of the two diel genes whose expression was investigated by fluorescent *in situ* hybridisation.** Adult worms were fixed for FISH at ZT 4.5. Even though *PPIase* peaks during the dark phase it is still expressed during the day enabling transcripts to be localised in cells.
