# Supplementary material for: Daily rhythms in gene expression of the human parasite Schistosoma mansoni
Source: BMC Biol. 2021 Dec 2;19:255. doi: 10.1186/s12915-021-01189-9 (PMC8638415; doi:10.1186/s12915-021-01189-9)
Supplement: Supplementary file 3 — Additional file 3: Supplementary information 1. SmKI-1, a Bovine Pancreatic Trypsin Inhibitor/Kunitz protease inhibitor domain protein. Supplementary information 2. Clock gene protein structure. [file 12915_2021_1189_MOESM3_ESM.docx]

**Daily rhythms in the transcriptomes of the human parasite *Schistosoma mansoni***

Kate A. Rawlinson1^🖂^, Adam J. Reid1, Zhigang Lu1, Patrick Driguez1,2, Anna Wawer3, Avril Coghlan1, Geetha Sankaranarayanan1, Sarah Kay Buddenborg1, Carmen Diaz Soria1, Catherine McCarthy1, Nancy Holroyd1, Mandy Sanders1, Karl Hoffmann3, David Wilcockson3, Gabriel Rinaldi1, Matt Berriman1^🖂^

1. Wellcome Sanger Institute, Wellcome Genome Campus, Hinxton, UK.

2. King Abdullah University of Science and Technology, Thuwal, Makkah, Saudi Arabia

3. Institute of Biological, Environmental, and Rural Sciences, Aberystwyth University, Aberystwyth, UK

Corresponding authors: Kate Rawlinson (kr16@sanger.ac.uk) and Matt Berriman (mb4@sanger.ac.uk)

**Legend**

**Additional file 3:** **Supplementary information 1** SmKI-1, a Bovine Pancreatic Trypsin Inhibitor/Kunitz protease inhibitor domain protein**. Supplementary information 2** Clock gene protein structure.

**Supplementary information 1**

**Smp_307450 - SmKI-1, a Bovine Pancreatic Trypsin Inhibitor/Kunitz protease inhibitor domain protein**

**
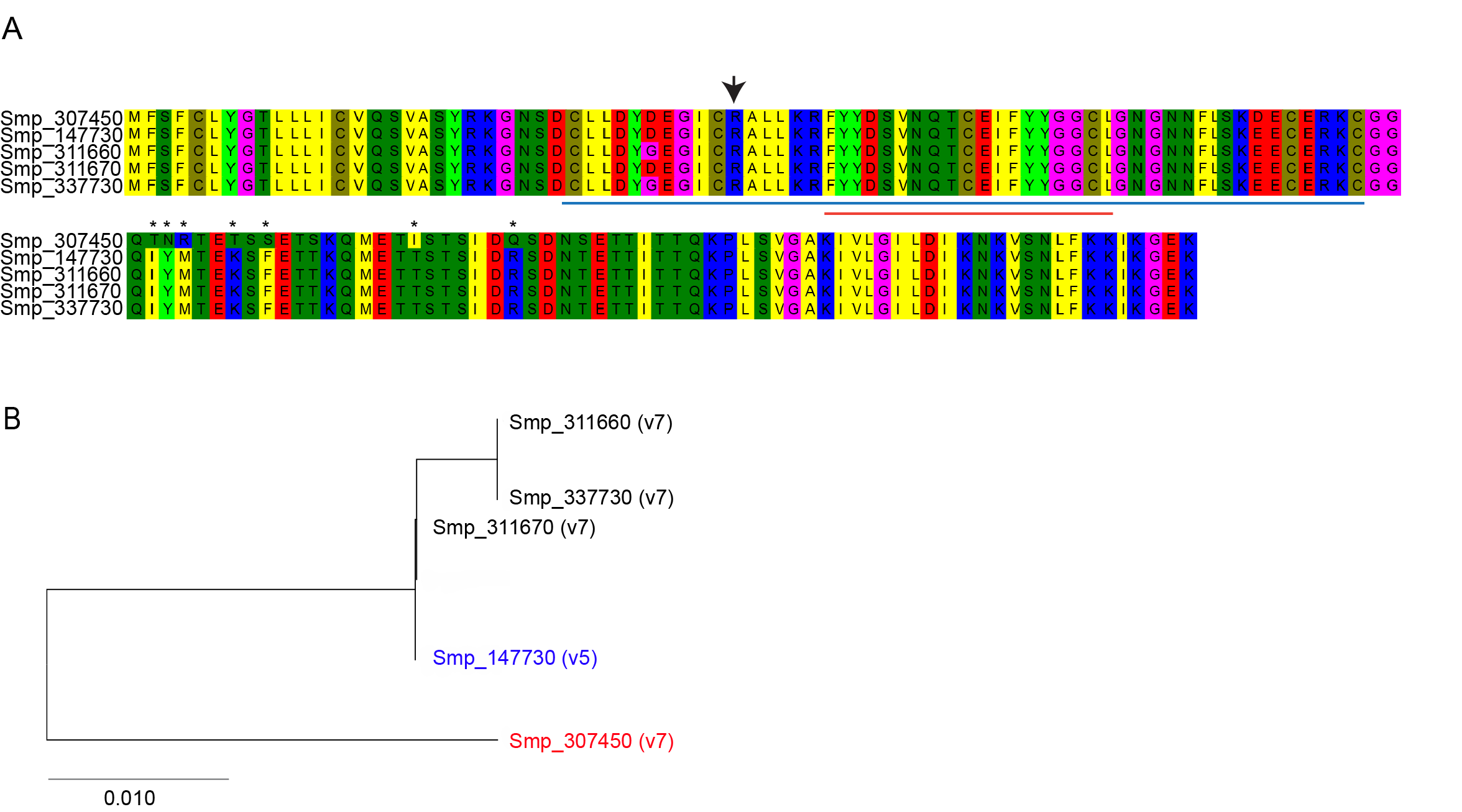
**

**Supplementary Information 1 Figure A)** ClustalW alignment and **B)** Neighbour joining tree (MEGA 7) of Smp_307450, Smp_311660, Smp_311670, Smp_337730 (v7 of genome) and Smp_147730 (v5). Kunitz domain (blue line), reactive P_1_ site (black arrow head), and the Kunitz family signature (red line). * amino acid differences between Smp_307450 and Smp_147730.

Smp_307450 was the diel gene with the highest amplitude in male worms. It was previously identified as Smp_147730 in version 5 of the genome. Smp_147730 has been split into 4 genes in version 7; Smp_307450, Smp_311660, Smp_311670, Smp_337730. Aligning Smp_147730 (from UniProt) to Smp_307450, Smp_311660, Smp_311670, Smp_337730, shows that they are all very similar; containing the Kunitz domain, with its six conserved cysteine residues [34], the Kunitz family signature [35], and the same amino acid residue at the reactive P_1_ site [36]. The P_1_ site is the major determinant of the specificity of protease recognition by Kunitz inhibitors; typical trypsin inhibitors contain Arg (R) or Lys (K) [34]. Smp_307450 differs from Smp_147730 at seven sites, but the conserved Arg at the P_1_ site indicates that Smp_307450 is potentially a trypsin inhibitor.

**Supplementary information 2**

**Clock gene protein structure**


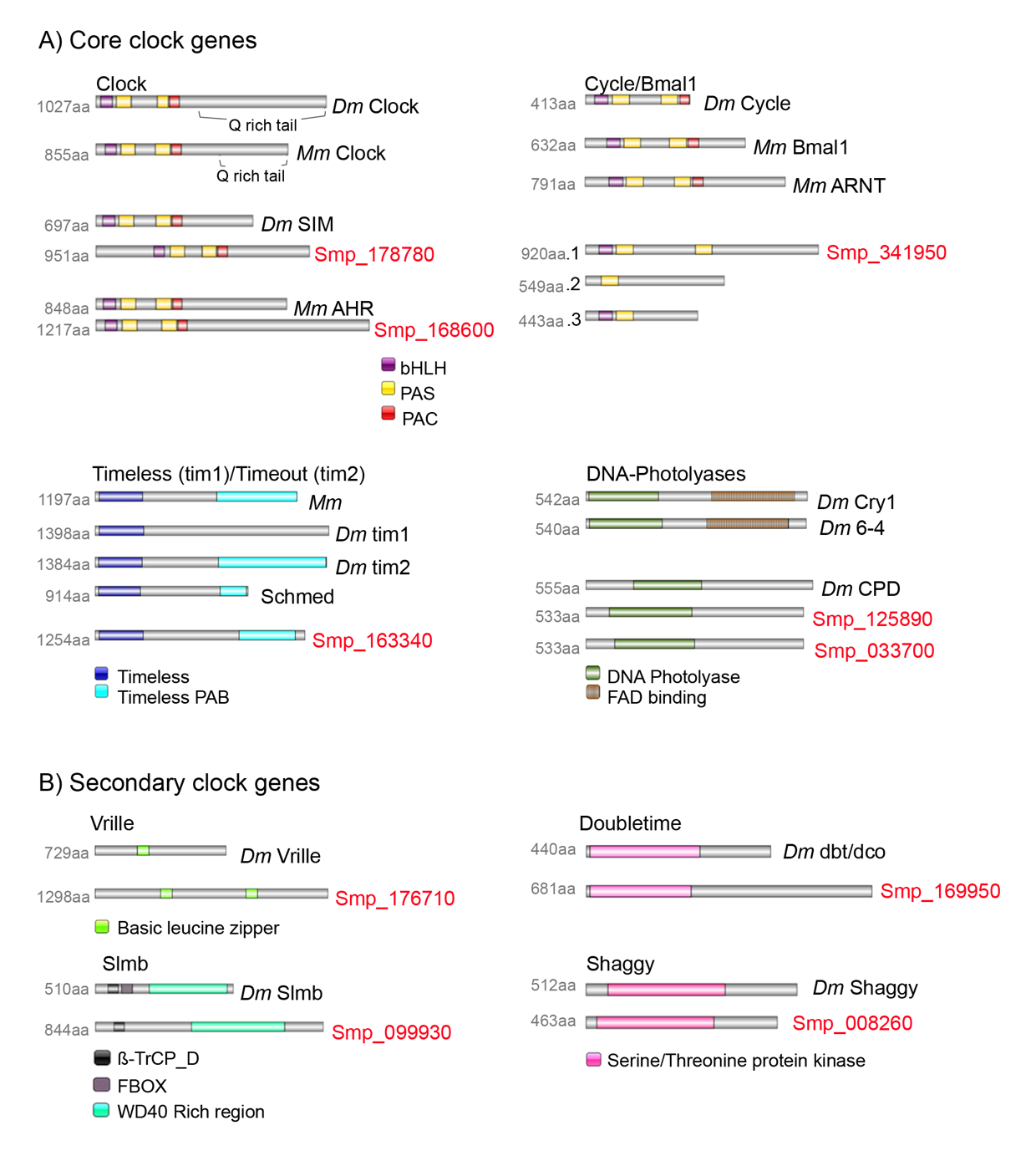


**Supplementary Information 2 Figure. Secondary structural features of putative circadian clock proteins in *Schistosoma mansoni*.**  **A)** Core clock components and **B)** secondary clock components. Note the missing Q-rich tail in SIM/ AHR and the missing FBOX domain in *Slmb* homolog. Amino acids are indicated on the left and names are shown on the right.

***Core clock genes***

**Basic-helix-loop-helix-PAS transcription factor family**

The basic-helix-loop-helix-PAS transcription factor family contains the domains bHLH, PAS, PAC [42]. CLOCK and CYCLE/BMAL1 are members that are known to be involved in the animal circadian clock [43]. CLOCK, in addition to the family-wide domains, has a characteristic poly-Q (polyglutamine) domain (SI Fig.2). Shortening of the poly-Q domain results in impairment of transcription activity of clock [44,45]. This domain is missing in our BLASTP hits (Smp_168600, Smp_178780 and Smp_341950) (SI Fig.2), which instead clustered within the AHR, SIM, and ARNT clades respectively (Additional file 2; Supplementary figure 11). The identified ARNT homolog in *S. mansoni* (Smp_341950) also lacked the PAC motif in all three of its splice variants (SI Fig.2). The PAC motif contributes to PAS binding [46] and has been concluded to be a part of the PAS domain [47].

**Timeless (tim1)/ Timeout (tim2)**

*tim1* and *tim2* are paralogous genes [48], with *tim1* duplicating from *tim2* at the time of the Cambrian explosion [49]. Like mouse and fly *tim2,* but unlike fly *tim1,* *S. mansoni,* and the free-living flatworm *S. mediterranea,* have *tim2* orthologues that contain a PAB domain at their C- terminus (SI Fig.2). In humans, the PAB domain is known to be involved in DNA repair by binding to a DNA repair enzyme Poly ADP-ribose polymerase 1 (PARP-1) [50].

**Cryptochrome/Photolyase family**

The FAD binding domain prominent in DNA Photolyases and Cryptochromes is absent in *S. mansoni’s* CPD photolyases (SI Fig.2).

***Secondary clock genes***

We identified an *S. mansoni* homolog of *Vrille* *(vri)* (Smp_176710). Whereas the insect model only has one Basic Leucine Zipper (bzip) domain, the *S. mansoni* homolog appeared to have a double bzip domain (SI Fig.2). The Activating Transcription Factor 2 (ATF-2) in *C. elegans* is the only current double bzip domain protein studied in literature [51, 52](Additional file 2; Supplementary figure 12). Our hit for *doubletime* (Smp_169950) clustered with its mammalian homolog glycogen synthase kinase GSK3 (Additional file 2; Supplementary figure 12). Neither SMART nor Pfam could identify an FBOX in our *slmb* homolog (SI Fig.2). However, our hit (Smp_099930) was clustered with *lin-23*, the *C. elegans* homolog of *slmb* (Additional file 2; Supplementary figure 12).
